# The Intricate Mechanism of Nitric Oxide Synthase

**DOI:** 10.1002/jcc.70448

**Published:** 2026-06-23

**Authors:** Per E. M. Siegbahn

**Affiliations:** ^1^ Department of Organic Chemistry, Arrhenius Laboratory Stockholm University Stockholm Sweden

## Abstract

The biological formation of NO is performed by nitric oxide synthase. For the first half of the mechanism, a consensus understanding has been reached, but not for the second half. Several attempts to reach a mechanism in agreement with experimental observations have been reported, but without success. Therefore, another study of that half‐reaction has been made here. The main difficulties have been to explain the observed non‐stoichiometric action of the cofactor H_4_B, and also why only one outside reduction from NADP is enough. A key new finding here is that the first step involves a protonation of the proximal cysteine of the heme. O_2_ then binds to iron and forms O_2_H with a proton from the NOH ligand of the substrate NHA. A long‐range electron transfer from H_4_B leads to the formation of the heme bound H_2_O_2_, which is followed by a dissociation of the O—O bond of H_2_O_2_. The last step studied here was a release of NO and a reverse electron transfer back to H_4_B. The results are in very good agreement with experimental observations, which is in line with previous findings for other redox enzymes using the present methodology.

## Graphical abstract

The mechanism for NO formation by Nitric Oxide Synthase (NOS) has been studied using accurate DFT methods. A long‐range electron transfer from the cofactor H_4_B leads to formation of H_2_O_2_.

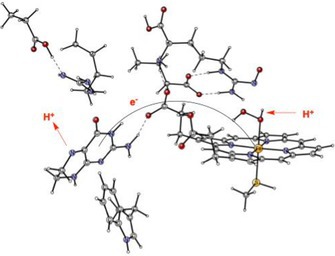

## 1 Introduction

Nitric Oxide Synthase (NOS) catalyzes the production of NO from L‐arginine. NO has several important roles in the human body, such as acting in protection against bacterial and inflammatory conditions [1, 2, 3, 4].

**FIGURE 1:**
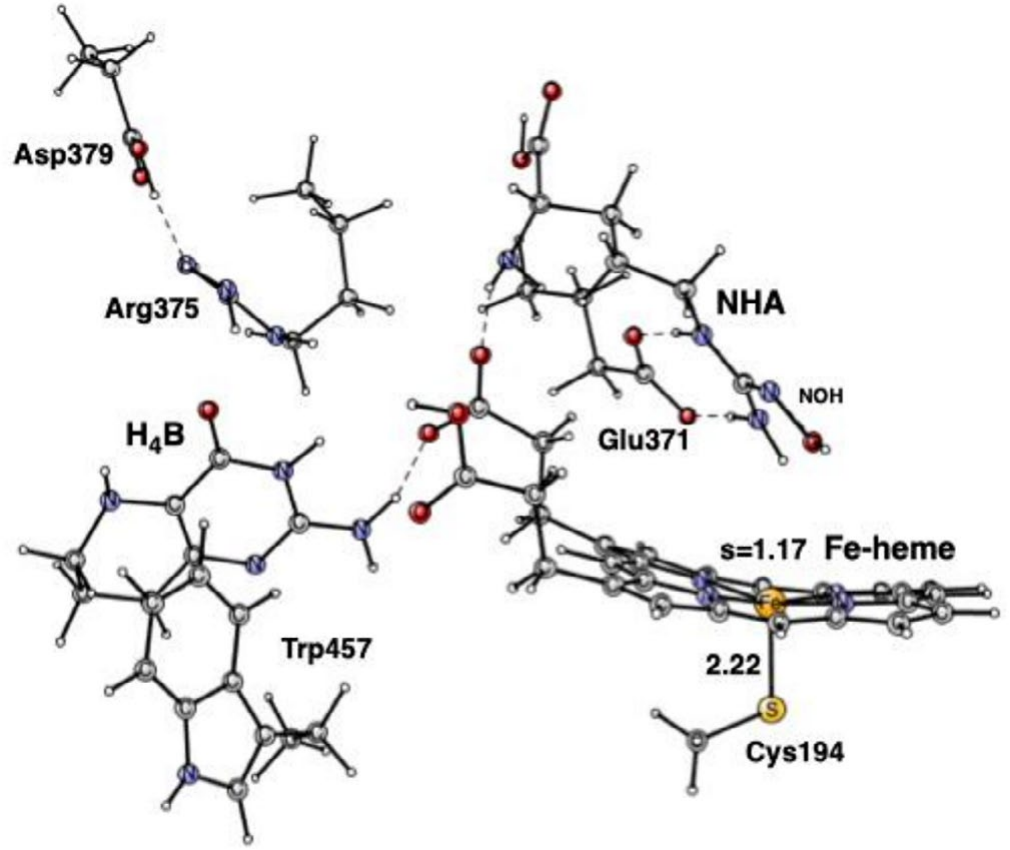
The active site of nitric oxide synthase (NOS) based on the PDB structure 1NOD [5]. The model is taken from a previously optimized structure in a study for a much larger model than used here [6]. The charge of the model is −1 and the total spin is a triplet. Iron is Fe(III).

In the active site of NOS there is a P450‐like Fe‐heme with a proximal cysteine ligand, see Figure 1. There is also an important cofactor 5,6,7,8‐tetrahydrobiopterin (H_4_B). The formation of NO involves two half‐reactions. In the first half, the substrate L‐arginine is transformed to C‐dehydroxy‐C‐amino‐asparagine (NHA). There is essential agreement on the mechanism for that step involving a ferryl‐oxo species similar to Compound I of P450 [7, 8, 9, 10, 11]. Several quantum chemical studies have also been done on the mechanism for the second half‐reaction of NOS, where the final product NO is formed from NHA. However, a clear consensus has not been reached.

A thorough review on the experimental information and its interpretation for the second half‐reaction has been written [6], and the reader is referred to that review for more information. EPR cryoreduction experiments [9, 10] have been interpreted to show that Compound I is not formed in this half‐reaction. Still, since the experimental information is not entirely clear, most quantum chemical studies performed [12, 13, 14, 15, 16, 17, 18], have been built on the formation of that species.

In the present study of the second half‐reaction, it has been investigated whether the formation of Compound I can be avoided as suggested experimentally. The mechanism obtained has similarities to the one described in the review [6], but with some important differences. The present paper is part of a series in which mechanisms of redox enzymes are studied using the same methodology [19, 20, 21, 22, 23]. So far, there is excellent agreement with all available experiments.

## 2 Methods

The methods used here are essentially the same as the ones used in many previous studies [19, 20, 21, 22, 23]. Density functional Theory (DFT) is used with the B3LYP functional [24]. Instead of the original 20% exact exchange, 15% is used. The choice is based on experience on many redox enzymes [19, 20, 21, 22, 23]. DFT is used in the unrestricted form. The basis set used for the geometry optimizations, for the Hessian calculations and for the solvation effects [25] is lacvp*. For the final point energies, a larger basis set is used with cc‐pvtz(−f) for all atoms except iron, where lacv3p + is used. For the dispersion effects, the empirical D2 correction was used [26]. For solvation effects a dielectric constant of 4.0 was used [25]. The cluster model was used [27, 28] with the fixed atoms given in the Supporting Information. In support for using a cluster model, the dielectric effects of all steps in the mechanism are quite small, indicating only small long‐range effects. The only goal with fixing atoms is to keep the structure as close as possible to the X‐ray structure. To achieve this, without disturbing the chemistry, the model must be big enough and the fixed atoms should be distributed in the periphery of the model. There should be no other effect on the mechanism by the fixing of some atoms. The Jaguar [25] and Gaussian [29] programs were used for the calculations.

An alternative to the cluster model could be to use QM/MM, suggested in 1976 [30]. While the cluster model has been widely tested on the mechanisms of many redox enzymes with excellent results compared to experiments, there are very few examples of using QM/MM for the mechanisms of these systems. The experience has so far not been positive. QM/MM was used to study the mechanism for Photosystem II, with poor results [31] compared to experiments that were done afterwards. In contrast, the cluster model done at the same time as the QM/MM study, gave excellent results for both structure and mechanism [19]. A QM/MM study on nitrogenase did not reach a correct mechanism either [32], in contrast to a cluster model study [20, 21]. Another example, where QM/MM was compared to a cluster study, with the same outcome was the mechanism for tyrosinase [33]. The idea behind the QM/MM method is interesting, but it is apparently difficult to use properly. One major problem with using QM/MM is that it is cumbersome to use. Mechanisms of this type require very many different structures, which need to be compared. In the above studies, only a few different structures were compared. Another problem is that QM/MM requires extensive sampling with sometimes several 100 different starting structures, which becomes very expensive combined with high accuracy DFT methods. Sampling is not needed for the cluster model, once the lowest energy state is found.

It is well known that for obtaining accurate redox potentials, a large model is required to take care of the surrounding, because there is a change of charge and the coulomb effect stretches far out. The cluster model is in general too small for that purpose. However, an important finding in previous studies is that for the energy of proton coupled redox potentials (PCET), there is no need for a large model since there is no change of charge. The only redox transitions in the present study are of PCET type, so no need for a large model. The energy for the step when an outside electron enters is obtained using thermodynamic data [34, 35]. The solvation free energy of a proton is a fundamental quantity in solution‐phase thermodynamics. A widely adopted experimental value for the hydration energy of a proton is −265.9 kcal/mol [36]. However, this value must be corrected to the standard state (1 M concentration), which contributes a correction factor of +1.9 kcal/mol, yielding a standard state solvation free energy, *ΔG*°(H^+^) = −264.0 kcal/mol. To connect this to a typical computational framework, the translational entropy of a gas‐phase proton must be considered. Analytical frequency calculations assign a value of −6.3 kcal/mol for this contribution. Incorporating this gives a composite value of −270.3 kcal/mol for the standard state (pH = 0). For biochemical applications, a further correction to pH 7 is required. Applying the term 1.364 × pH (which equals 9.548 kcal/mol at pH 7) results in a free energy of −279.9 kcal/mol for the reaction of adding a proton to an aqueous solution. Consequently, the free energy cost for deprotonation (i.e., removing a proton) is the opposite, +279.9 kcal/mol. This thermodynamic cycle is directly related to redox potentials. The absolute potential of the Standard Hydrogen Electrode (SHE) is 4.281 V. Therefore, a redox couple with a reported potential of −0.3 Versus SHE for NADPH [37] has an absolute potential of 4.281–0.3 = 3.981 V. Using the conversion factor 1 V = 23.0605 kcal/mol, this absolute potential corresponds to a free energy change of 91.8 kcal/mol. The sum is 371.6, which should be subtracted from the calculated value for the energy of adding (H^+^, e^−^) to the metal complex in obtaining the driving force.

The by far largest entropy effect occurs when O_2_ enters the mechanism. Following the procedure in previous papers, there is at that point a loss of translational entropy of 10.8 kcal/mol, calculated from a particle in a box. Other entropy effects are by experience very small [20, 21, 22, 23]. The calculated Hessians cannot be used for the entropy effects since some atoms are fixed. It can be added that there is often an exchange of energy between entropy and enthalpy, while the free energy is not changed [38]. This means that it is often wrong to take a calculated value without entropy and add measured entropies. The present results are, therefore, better compared to free energies since entropy corrections are generally very small, with the exception mentioned above.

## 3 Results

The present study on the second half‐reaction of the mechanism for nitric oxide synthase (NOS) starts at the structure shown in Figure 1 with the central Fe‐heme complex having Cys194 at its proximal site. The substrate NHA and the cofactor H_4_B are included in all calculations done here. H_4_B has 1H—bond to Arg375, and 2H—bonds to one of the propionates of the heme, which hold H_4_B in place. One of the propionates is unprotonated since it has strong hydrogen bonds. The other one is protonated. Glu371 has two strong hydrogen bonds to the substrate NHA, which hold it in place. The model also has some peripheral amino acids included, Asp379, and Trp457, which do not directly participate in the mechanism. The NO‐group of NHA is protonated and the iron is Fe(III). The starting coordinates were taken from the 1NOD X‐ray structure [5] as further optimized for a very large model [6]. The charge of the model is −1 and the spin is a triplet. There are two main spins, one on iron, making it Fe(III), and the other one on the NOH ligand of NHA. The cysteine has essentially no spin. The triplet and singlet are nearly degenerate, as expected with two rather distant spins. More surprising is that also the quintet is very close in energy. In that state, iron is Fe(II) and the cysteine has a high spin with −0.52, which has some implications for the first step of the mechanism, described below.

In the first step of the mechanism, the active site is reduced by the outside reductant NADPH. The electron reduces iron from Fe(III) to Fe(II). In that process, a proton is taken up by the sulfide of Cys194. The state is a doublet, but the quartet is very close in energy with three rather distant spins. The Fe‐S bond distance increases from 2.22 Å to 2.97 Å. The reduction step with a proton on the cysteine is exergonic by −8.8 kcal/mol using a redox potential of −0.3 V for NADPH [36]. The low‐lying quintet of the ground state plays a role here. The present study disagrees significantly with a previous study [6], where there was no proton uptake on the sulfide but instead one to a carbon of the heme [6]. The present calculations very strongly favor the uptake to the cysteine by 34 kcal/mol (lacvp*). It is clear that with such a large error in the first step, the previously suggested mechanism has to be discarded.

**FIGURE 2:**
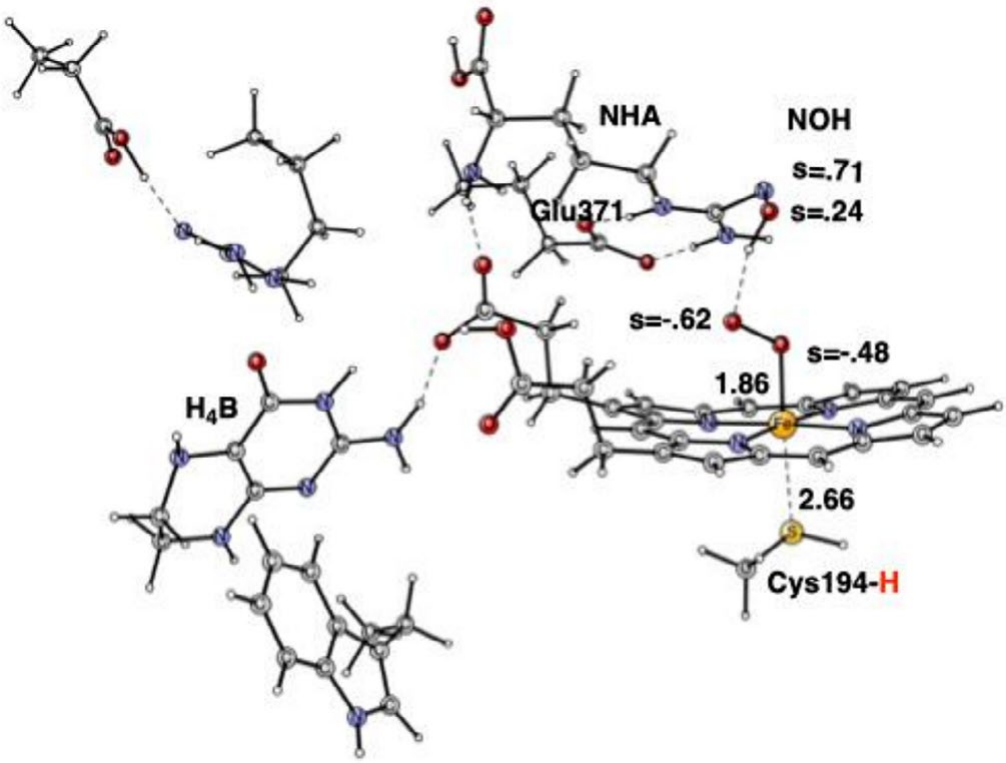
The structure and spin distribution obtained when O_2_ is added to iron. Iron becomes Fe(III) (*s* = 1.19). The structure is termed “O_2_” in the diagram below. The charge of the model is −1 and the total spin is a doublet I.

In the next step of the present mechanism, O_2_ binds to the iron of the heme. The enthalpic binding energy is 8.6 kcal/mol, but there is also a large loss of translational entropy of 10.8 kcal/mol, making the free energy of binding slightly endergonic with 2.2 kcal/mol. Iron remains Fe(III) with a spin of 1.19. The spins on O_2_ are −0.62 and −0.48 indicating an O_2_•^−^ radical. There is not yet any spin on H_4_B. The total spin is a doublet but the quartet is rather close in energy at +5.5 kcal/mol (lacvp*). The NOH ligand of NHA forms a hydrogen bond with O_2_ and has a spin of 0.95, see Figure 2.

**FIGURE 3:**
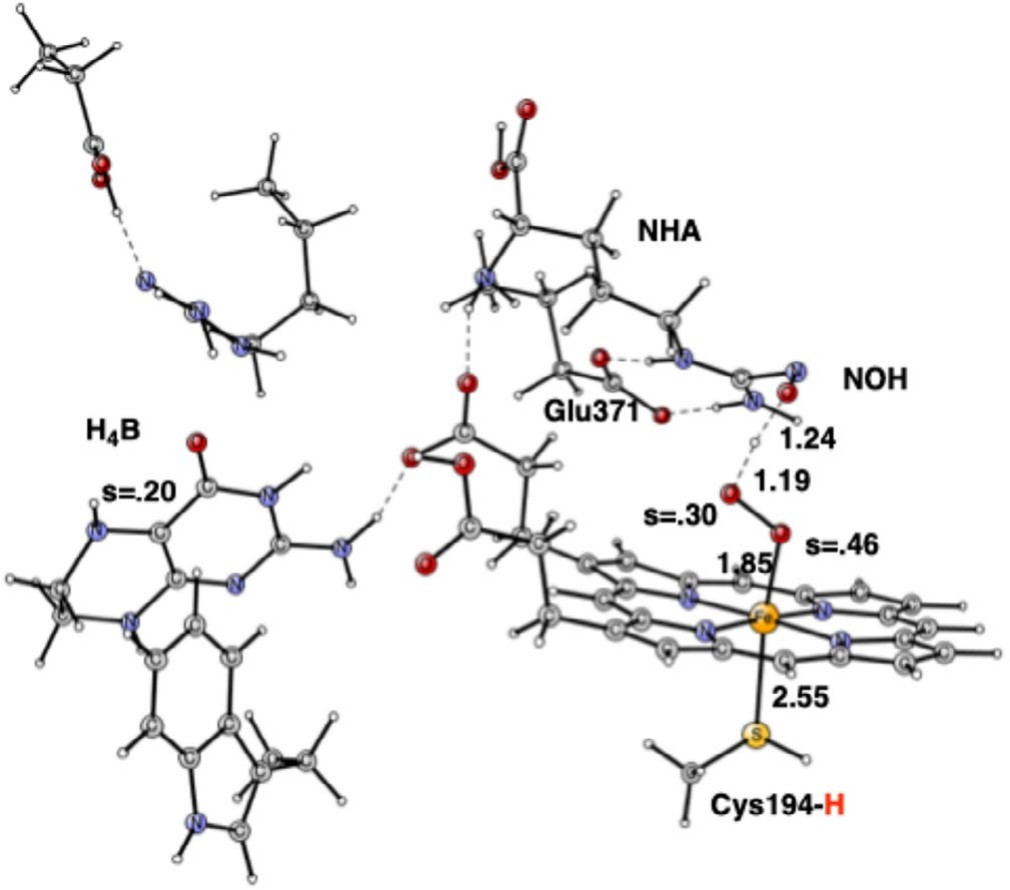
The TS structure and spin distribution obtained when a proton is moved from NOH to O_2_. Notably, H_4_B has obtained some spin. Iron has a mixture of Fe(II) and Fe(III) (*s* = 36). The charge of the model is −1 and the total spin is a doublet.

The step above is followed by a proton transfer between NOH and O_2_. The TS is shown in Figure 3. There are two almost equal O‐H distances of about 1.2 Å, which is typical for this type of TS. A rather surprising finding is that the spin on iron decreases from 1.19 for the reactant, typical for Fe(III), down to 0.36, which indicates a mixture with Fe(II) low spin. The spin goes from iron, both to O_2_ and to NOH. There is also some spin on H_4_B for the first time, with about 0.2. The barrier is only 2.5 kcal/mol, where solvation has a lowering effect of −4.9 kcal/mol. The step is exergonic by 7.7 kcal/mol. The product O_2_H has a strong bond to iron with a distance of 1.78 Å. Iron is Fe(III) again with a spin of 1.01. The spin on H_4_B and NO are down to zero.

**FIGURE 4:**
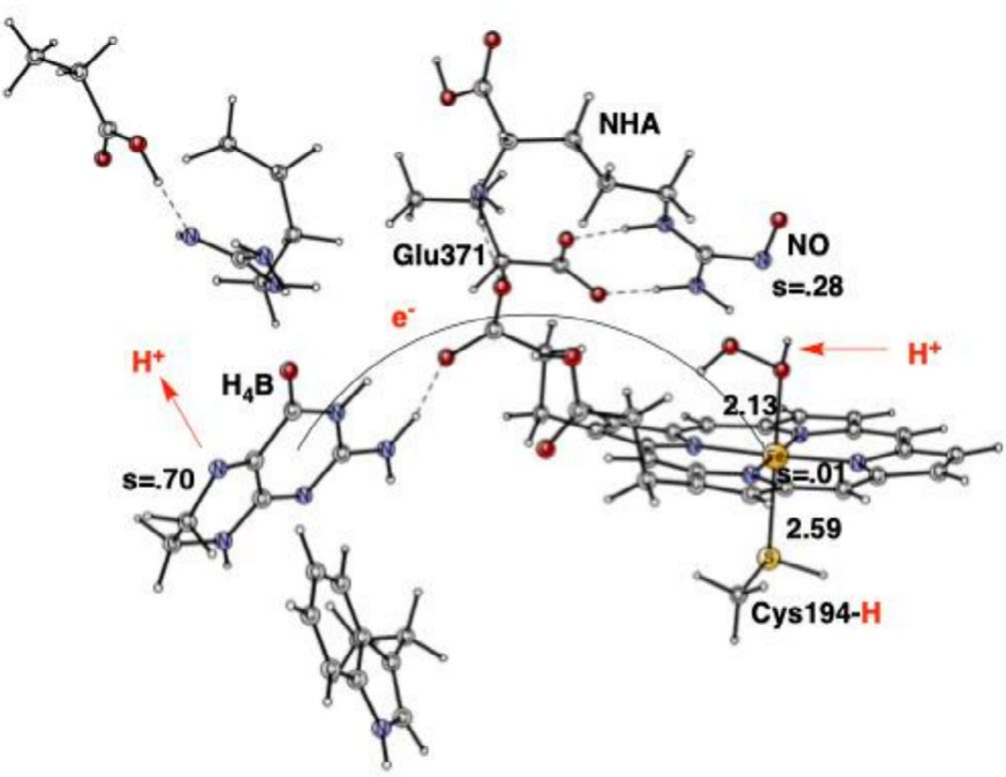
The structure and spin distribution obtained for H_2_O_2_. An electron has moved from H_4_B to iron, forming low spin Fe(II). The protonations are simultaneously equilibrated, leading to a protonation of O_2_H to H_2_O_2_ and a deprotonation of H_4_B. The energy is 13.2 kcal/mol above the resting O_2_H state. The structure is termed “H_2_O_2_” in the diagram below. The charge of the model is −1 and the total spin is a doublet.

In the next step of the mechanism for NOS there is an electron transfer from H_4_B to iron, followed by an equilibration of the complex with the water medium, which leads to a deprotonation of a nitrogen of H_4_B and a protonation of O_2_H to H_2_O_2_. The structure is shown in Figure 4. The step is rather strongly endergonic by 13.2 kcal/mol, but it is unclear if H_2_O_2_ is actually in a minimum, see below. H_4_B has a spin of 0.7 and NOH has one of 0.3. There is no spin on iron, indicating Fe(II) with low spin coupling. The high spin state of Fe(II) on the quartet surface is close in energy, only 1.5 kcal/mol higher.

**FIGURE 5:**
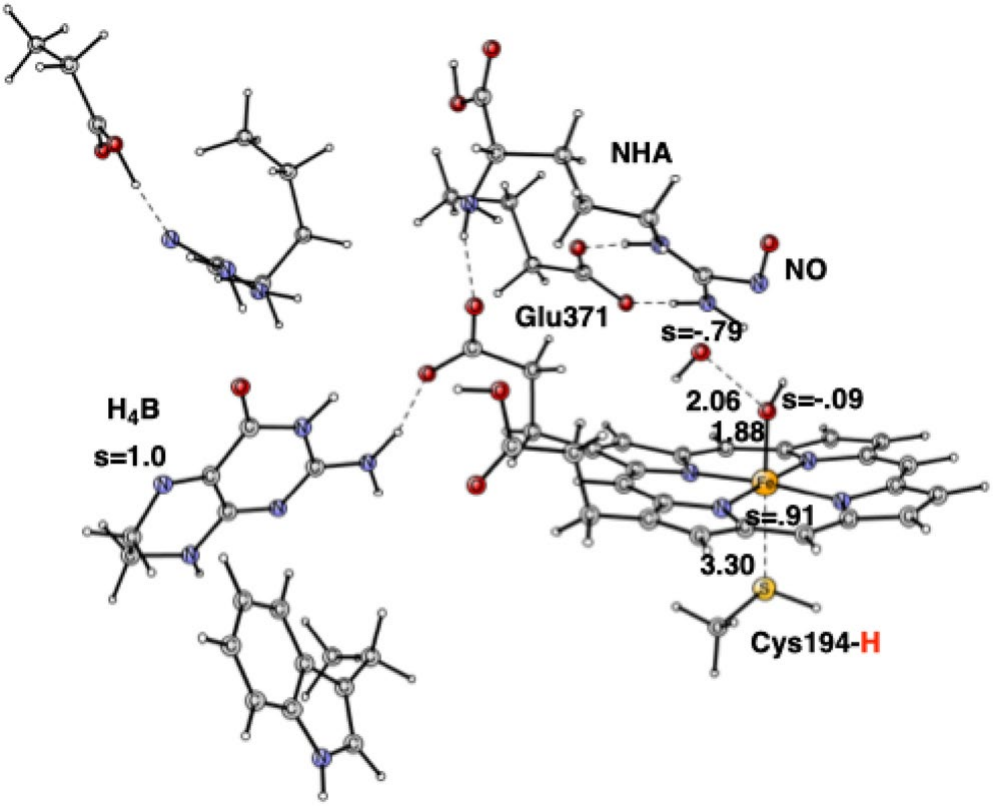
The optimized TS structure and spin distribution obtained when the O—O bond of H_2_O_2_ is broken. Iron is Fe(III) and H_4_B is a radical with spin 1.0. The charge of the model is −1 and the total spin is a doublet.

After H_2_O_2_ is formed, the O—O bond is broken. The optimized TS, at the lacvp* level, for O—O bond cleavage is shown in Figure 5 and is quite surprising. One of the OH groups formed binds to iron, as expected, but the other one has a spin of 0.79, so the radical is almost formed. The spins on the atoms of NO are zero. The high energy cost for creating an OH—radical is partly compensated by the strong Fe—O bond formed. The Fe—O bond distance has decreased from 2.13 Å to 1.88 Å at the TS. The spin on iron has increased from 0.01 to 0.91, which is typical for Fe(III). The TS energy was actually found to be lower than the one for H_2_O_2_, when solvation and zero‐point effects were added. Therefore, it is not clear if the calculated barrier point should be regarded as a TS, but in the energy diagram it is still termed “TS”. The free OH‐radical, which is in a very shallow minimum with hardly any barriers, can obviously not live for long, but will move to form a C—OH bond on NHA. This leads to an automatic loss of NO. The formation is very exergonic, leading to a product which is −79.7 kcal/mol lower than the resting O_2_H state. Iron is still Fe(III). The spins on the atoms of NO are now zero.

**FIGURE 6:**
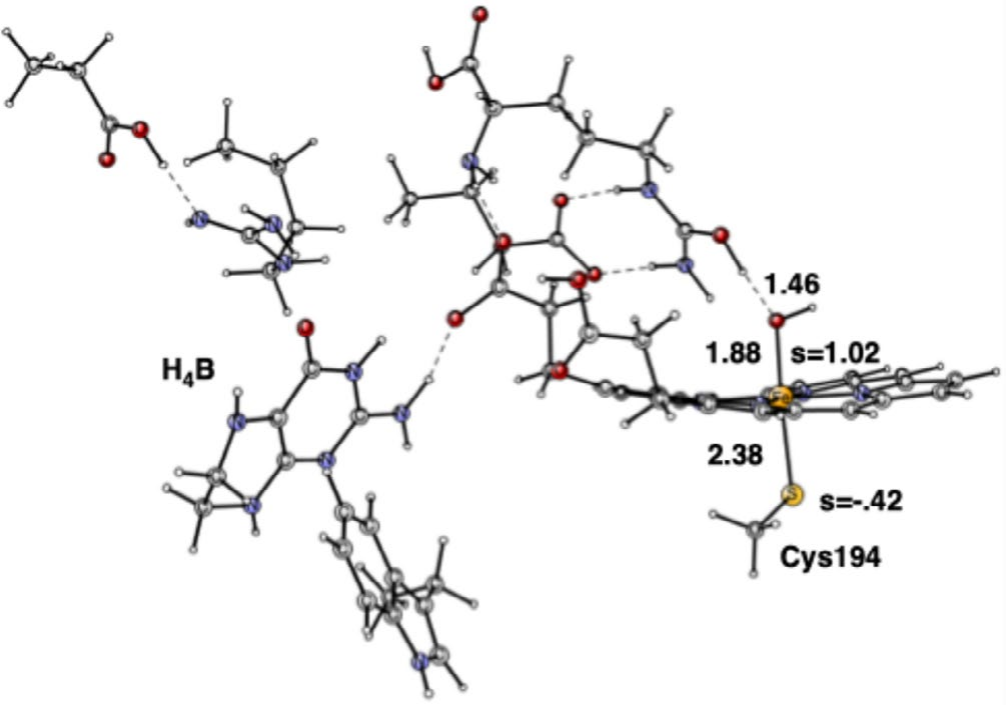
The final Fe‐OH structure in the calculated mechanism. The charge of the model is −1 and the total spin is a triplet. Iron is Fe(III) (*s* = 1.02).

In the next step, the electron goes back to H_4_B and the protonations are equilibrated, which in this case leads to a protonation of H_4_B and a deprotonation of Cys194. A very strong hydrogen bond with a distance of only 1.46 Å to FeOH can be noted. The step is exergonic by 7.3 kcal/mol., and leads to the structure shown in Figure 6.

It should be noted that the catalytic cycle is not yet closed since the release of water was found to be quite endergonic. Instead, release of water has to wait until the product citrulline is released and the next NHA becomes bound. That step is too difficult to calculate, since it requires a detailed modeling of the water medium simultaneously with a large quantum mechanical modeling of the enzyme active site, which is not possible to describe with the present methods.

**FIGURE 7:**
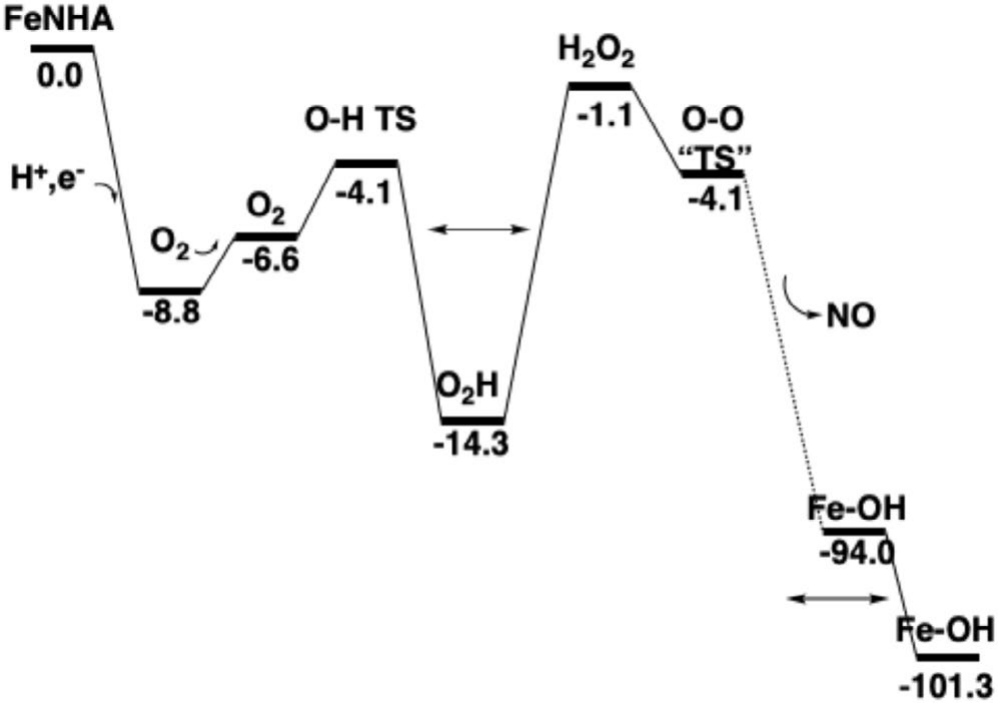
Energy diagram for the mechanism of NOS. The double arrow signifies a proton and electron transfer within the model involving H_4_B.

The energy diagram for the process ending in the formation of Fe—OH is shown in Figure 7. There is very good agreement with the sparse experimental information, where only the reactant structure in Figure 1 has been characterized. The present conclusions also agree with the available spectroscopic information and suggestions. Most importantly, only one outside electron is needed for the catalysis, and the electron from H_4_B is not stoichiometric. A clear role for the H_4_B cofactor has been found with a key electron transfer step when H_2_O_2_ forms and becomes activated. The value for the rate‐limiting barrier in the diagram is 13.2 kcal/mol in reasonable agreement with the experimental value of 16.4 kcal/mol [36]. A calculated barrier that is smaller than the experimental one is unusual. Therefore, it is possible that the final step, not calculated here, is rate‐limiting.

Many different mechanisms for the second part of the NOS mechanism have been suggested. The similarity of the NOS structure with that of P450 has led to suggestions for a similar mechanism for NOS with the formation of compound I. However, its formation requires more than one outside electron in contrast to the experimental findings.

Another type of mechanism was briefly investigated further here. In that mechanism, the iron bound O_2_ forms a bond to the guanidium carbon of NHA leading to a tetrahedral intermediate, which was suggested in the first study of the NOS mechanism [15]. The attack of O_2_ on the guanidium carbon was studied with a starting structure where O_2_ is bound to Fe(III), see Figure 2. H_4_B is neutral in its non‐radical form. There is one spin on NOH and Cys194 is protonated after the reduction by NADPH. The charge of the model is −1 and the total spin is a doublet. To study this step, the C—O_2_ bond was shortened in steps. Already at a C—O distance of 2.10 Å, the energy is very high, and it appears to increase even more for shorter C—O distances. It is clear that the mechanism is not competitive with the preferred mechanism described here.

## 4 Summary

The mechanism for Nitric Oxide Synthase (NOS) has been studied with methods that have been used for many other redox enzymes before, with excellent results [19, 20, 21, 22, 23]. Also the mechanism given here for NOS agrees very well with all observations from spectroscopy. The mechanism starts with a proton coupled reduction by NADPH. The electron reduces Fe(III) to Fe(II) and the proton goes to the sulfur of Cys134, which is the proximal ligand on the heme. The protonation of Cys134 markedly increases its bond distance to iron, which leaves iron essentially 4‐coordinated. That protonation is an aspect of the NOS mechanism, which has not been suggested before. In the most thorough earlier study [6], a protonation of a carbon of the heme was suggested instead, which is here shown to be more than 30 kcal/mol higher in energy than protonating Cys134.

In the following step, O_2_ becomes bound, in a slightly endergonic step. O_2_H is formed by obtaining a proton from the NOH ligand of NHA. In connection with the formation of H_2_O_2_, an electron is transferred from the cofactor H_4_B to iron and Fe(III) is reduced to Fe(II), see Figure 4. The electron transfer is accompanied by a proton equilibration, which leads to a protonation of O_2_H and a deprotonation of a nitrogen on H_4_B. H_4_B is at that stage a neutral radical.

The key step of the mechanism, where the O—O bond is cleaved, is quite surprising. Rather than starting to form the expected C—O bond to the guanidium carbon, an OH—radical is formed. Therefore, C—O bond formation does not drive O—O bond cleavage. The OH—radical will only afterwards form the C—O bond with almost no barrier in a very exergonic step. In connection with the formation of the C—OH bond, the product NO is released, which leads to the final structure studied here which has a Fe—OH bond. For completing the mechanism, water should be released, but that step is quite endergonic. Water will instead leave in the step where a new reactant enters. That step is very difficult to model and has not been studied here.

An important agreement with experiments concerns the number of electrons needed from the NADPH reductant. Experiments have shown that only one outside electron is needed as found in the present study. In other studies, more outside electrons were needed in the suggested mechanisms [12, 13, 14, 15, 16, 17, 18]. Compound I is not formed in agreement with spectroscopic findings [9, 10]. Fully optimized transition states have been obtained leading to a rate‐limiting barrier of 13.2 kcal/mol in reasonable agreement with experiments [39].

## Funding

This work was supported by Stockholms Universitet.

## Supporting information


**Data S1:** jcc70448‐sup‐0001‐DataS1.docx.

## Data Availability

The data that supports the findings of this study are available in the Supporting Information of this article.

## References

[1] A. Berdeaux and A. Fundam , “Nitric Oxide – A Ubiquitous Messenger,” Fundamental & Clinical Pharmacology 7 (1993): 401–411. 8294080 10.1111/j.1472-8206.1993.tb01037.x

[2] A. V. Hall , H. Antoniou , Y. Wang , et al., “Structural Organisation of the Human Neuronal Nitric‐Oxide Synthase Gene (NOS1),” Journal of Biological Chemistry 269 (1994): 33082–33090. 7528745

[3] A. Hokari , M. Zeniya , and H. Esumi , “Cloning and Functional Expression of Human Inducible Nitric Oxide Synthase (NOS) cDNA From a Glioblastoma Cell Line A‐172,” Journal of Biochemistry 116 (1994): 575–581. 7531687 10.1093/oxfordjournals.jbchem.a124563

[4] F. H. Guo , R. H. de Raeve , T. W. Rice , D. J. Stuehr , F. B. J. M. Thunnissen , and S. C. Erzurum , “Continuous Nitric Oxide Synthesis by Inducible Nitric Oxide Synthase in Normal Human Airway Epithelium In Vivo,” Proceedings of the National Academy of Sciences of United States of America 92 (1995): 7809–7813. 10.1073/pnas.92.17.7809 PMC41235 7544004

[5] B. R. Crane , A. S. Arvai , R. Gachhui , et al., “Structure of Nitric Oxide Synthase Oxygenase Dimer With Pterin and Substrate,” Science 278 (1997): 425–431. 9516116 10.1126/science.279.5359.2121

[6] I. Shamovsky , G. Belfield , R. Lewis , et al., “Theoretical Studies of the Second Step of the Nitric Oxide Synthase Reaction: Electron Tunneling Prevents Uncoupling,” Journal of Inorganic Biochemistry 181 (2018): 28–40. 29407906 10.1016/j.jinorgbio.2018.01.009

[7] A. C. F. Gorren and B. Mayer , “Nitric‐Oxide Synthase: A Cytochrome P450 Family Foster Child,” Biochimica et Biophysica Acta 1770 (2007): 432–445. 17014963 10.1016/j.bbagen.2006.08.019

[8] J. Santolini , “The Molecular Mechanism of Mammalian NO‐Synthases: A Story of Electrons and Protons,” Journal of Inorganic Biochemistry 105 (2011): 127–141. 21194610 10.1016/j.jinorgbio.2010.10.011

[9] R. Davydov , A. Ledbetter‐Rogers , P. Martásek , et al., “EPR and ENDOR Characterization of Intermediates in the Cryoreduced Oxy‐Nitric Oxide Synthase Heme Domain With Bound l‐Arginine or *N* ^G^‐Hydroxyarginine,” Biochemistry 41 (2002): 10375–10381. 12173923 10.1021/bi0260637

[10] R. Davydov , J. Sudhamsu , N. S. Lees , B. R. Crane , and B. M. Hoffman , “EPR and ENDOR Characterization of the Reactive Intermediates in the Generation of NO by Cryoreduced Oxy‐Nitric Oxide Synthase From *Geobacillus stearothermophilus* ,” Journal of the American Chemical Society 131 (2009): 14493–14507. 19754116 10.1021/ja906133h

[11] C. C. Wei , Z.‐Q. Wang , C. Hemann , R. Hille , and D. J. Stuehr , “A Tetrahydrobiopterin Radical Forms and Then Becomes Reduced During *N* ^ω^‐Hydroxyarginine Oxidation by Nitric‐Oxide Synthase,” Journal of Biological Chemistry 278 (2003): 46668–46673. 14504282 10.1074/jbc.M307682200

[12] K.‐B. Cho , E. Derat , and S. Shaik , “Compound I of Nitric Oxide Synthase: The Active Site Protonation State,” Journal of the American Chemical Society 129 (2007): 3182–3188. 17319660 10.1021/ja066662r

[13] K.‐B. Cho , M. A. Carvajal , and S. Shaik , “First Half‐Reaction Mechanism of Nitric Oxide Synthase: The Role of Proton and Oxygen Coupled Electron Transfer in the Reaction by Quantum Mechanics/Molecular Mechanics,” Journal of Physical Chemistry B 113 (2009): 246–336. 10.1021/jp8073199 19072325

[14] K.‐B. Cho and J. W. Gauld , “Quantum Chemical Calculations of the NHA Bound Nitric Oxide Synthase Active Site: O_2_ Binding and Implications for the Catalytic Mechanism,” Journal of the American Chemical Society 126 (2004): 10267–10270. 15315438 10.1021/ja049186i

[15] K.‐B. Cho and J. W. Gauld , “Second Half‐Reaction of Nitric Oxide Synthase: Computational Insights Into the Initial Step and Key Proposed Intermediate,” Journal of Physical Chemistry. B 109 (2005): 23706–23714. 16375351 10.1021/jp054864o

[16] J. J. Robinet , K.‐B. Cho , and J. W. A. Gauld , “Density Functional Theory Investigation on the Mechanism of the Second Half‐Reaction of Nitric Oxide Synthase,” Journal of the American Chemical Society 130 (2008): 3328–3334. 18293966 10.1021/ja072650+

[17] S. P. de Visser and L. S. Tan , “Is the Bound Substrate in Nitric Oxide Synthase Protonated or Neutral and What Is the Active Oxidant That Performs Substrate Hydroxylation?,” Journal of the American Chemical Society 130 (2008): 12961–12974. 18774806 10.1021/ja8010995

[18] S. P. de Visser , “Density Functional Theory (DFT) and Combined Quantum Mechanical/Molecular Mechanics (QM/MM) Studies on the Oxygen Activation Step in Nitric Oxide Synthase Enzymes,” Biochemical Society Transactions 37 (2009): 373–377. 19290865 10.1042/BST0370373

[19] P. E. M. Siegbahn , “Structures and Energetics for O_2_ Formation in Photosystem II,” Accounts of Chemical Research 42 (2009): 1871–1880. 19856959 10.1021/ar900117k

[20] P. E. M. Siegbahn , “The Mechanism for N_2_ Activation in the E_4_ ‐ State of Nitrogenase,” Physical Chemistry Chemical Physics 25 (2023): 23602–23613. 37622205 10.1039/d3cp02851h

[21] P. E. M. Siegbahn , “The Final E_5_ to E_8_ Steps in the Nitrogenase Mechanism for Nitrogen Fixation,” Journal of Physical Chemistry B 128 (2024): 9699–9705. 39344806 10.1021/acs.jpcb.4c04331 PMC11472303

[22] P. E. M. Siegbahn , “The Mechanisms for Methane and Ammonia Oxidations by Particulate Methane Monooxygenase (pMMO),” Journal of Physical Chemistry B 128 (2024): 9699–9705. 38850249 10.1021/acs.jpcb.4c01807 PMC11194816

[23] P. E. M. Siegbahn , “The Nitrification Mechanisms for the P460 Enzymes,” Journal of Physical Chemistry B 129 (2025): 111–116. 39693510 10.1021/acs.jpcb.4c06537 PMC11726666

[24] A. D. Becke , “Density‐Functional Thermochemistry. III. The Role of Exact Exchange,” Journal of Chemical Physics 98 (1993): 5648–5652.

[25] A. D. Bochevarov , E. Harder , T. F. Hughes , et al., “Jaguar: A High‐Performance Quantum Chemistry Software Program With Strengths in Life and Materials Sciences,” International Journal of Quantum Chemistry 113 (2015): 2110–2142.

[26] S. Grimme , “Semiempirical GGA‐Type Density Functional Constructed With a Long‐Range Dispersion Correction,” Journal of Computational Chemistry 27 (2006): 1787–1799. 16955487 10.1002/jcc.20495

[27] P. E. M. Siegbahn and F. Himo , “The Quantum Chemical Cluster Approach for Modeling Enzyme Reactions,” Wiley Interdisciplinary Reviews: Computational Molecular Science 1 (2011): 323–336.

[28] F. Himo and S. P. de Visser , “Status Report on the Quantum Chemical Cluster Approach for Modeling Enzyme Reactions,” Communications Chemistry 5 (2022): 29. 36697758 10.1038/s42004-022-00642-2 PMC9814711

[29] Gaussian 09, Revision A.1 , M. J. Frisch , G. W. Trucks , H. B. Schlegel , et al., “Gaussian 09 Revision A.1. Gaussian Inc,” (2009).

[30] A. Warshel and M. Levitt , “Theoretical Studies of Enzymic Reactions: Dielectric, Electrostatic and Steric Stabilization of the Carbonium Ion in the Reaction of Lysozyme,” Journal of Molecular Biology 103 (1976): 227–249. 985660 10.1016/0022-2836(76)90311-9

[31] E. M. Sproviero , J. A. Gasco´n , J. P. McEvoy , G. W. Brudvig , S. Victor , and V. S. Batista , “Quantum Mechanics/Molecular Mechanics Study of the Catalytic Cycle of Water Splitting in Photosystem II,” Journal of the American Chemical Society 130 (2008): 3428–3442. 18290643 10.1021/ja076130q

[32] Y. Pangab and R. Björnsson , “The E_3_ State of FeMoco: One Hydride, Two Hydrides or Dihydrogen ?,” Physical Chemistry Chemical Physics 25 (2023): 21020–21036. 37522223 10.1039/d3cp01106b

[33] P. E. M. Siegbahn and T. Borowski , “Comparison of QM‐Only and QM/MM Models for the Mechanism of Tyrosinase,” Faraday Discussions 148 (2011): 109–117. 21322480 10.1039/c004378h

[34] A. V. Marenich , J. Ho , M. L. Coote , C. J. Cramer , and D. G. Truhlar , “Computational Electrochemistry: Prediction of Liquid‐Phase Reduction Potentials,” Physical Chemistry Chemical Physics 16 (2014): 15068–15106. 24958074 10.1039/c4cp01572j

[35] R.‐Z. Liao and P. E. M. Siegbahn , “Quantum Chemical Modeling of Homogeneous Water Oxidation Catalysis,” ChemSusChem 10 (2017): 4236–4263. 28875583 10.1002/cssc.201701374

[36] D. M. Camaioni and C. A. Schwerdtfeger , “Comment on Accurate Experimental Values for the Free Energies of Hydration of H^+^, OH^−^, and H_3_O^+^ ,” Journal of Physical Chemistry. A 109 (2005): 10795–10797. 16863129 10.1021/jp054088k

[37] M. B. Murataliev and R. Feyereisen , “Interaction of NADP(H) With Oxidized and Reduced P450 Reductase During Catalysis. Studies With Nucleotide Analogue,” Biochemistry 39 (2000): 5066–5074. 10819972 10.1021/bi992917k

[38] J. Åqvist , G. V. Isaksen , and B. O. Brandsdal , “Computation of Enzyme Cold Adaptation,” Nature Reviews Chemistry 1 (2017), 10.1038/s41570-017-0051.

[39] D. J. Stuehr , H. J. Cho , N. S. Kwon , M. F. Weise , and C. F. Nathan , “Purification and Characterization of the Cytokine‐Induced Macrophage Nitric Oxide Synthase: An FAD‐ and FMN‐Containing Flavoprotein,” Proceedings of the National Academy of Sciences of United States of America 88 (1991): 7773–7777. 10.1073/pnas.88.17.7773 PMC52385 1715579

