# Supplementary material for: The Intricate Mechanism of Nitric Oxide Synthase
Source: J Comput Chem. 2026 Jun 23;47(17):e70448. doi: 10.1002/jcc.70448 (PMC13291759; doi:10.1002/jcc.70448)
Supplement: Supplementary file 1 — Data S1: jcc70448‐sup‐0001‐DataS1.docx. [file JCC-47-0-s001.docx]

Supporting information:

The intricate mechanism of nitric oxide synthase

Per E. M. Siegbahn^*^

Department of Organic Chemistry, Arrhenius Laboratory, Stockholm University, SE-106 91, Stockholm, Sweden. Email:per.siegbahn@su.se

**xyz- coordinates for the structures discussed in the text.**

The structure in **Figure 1.** # means fixed atom

Energies: E= -4727.986360, solv = -0.109277, disp = -181.67 Z_0_ = 893.18

Fe1 128.1564040145 112.1420436431 95.4126393718

C2 132.0717288747 110.5634735117 91.6564897660

C3 131.5172489004 110.9846875583 90.2840969450

C4 132.6118008503# 111.2870028934# 89.2855001218#

O5 132.1125565594 111.3978760724 88.0314339308

H6 132.6006804743 109.6091561723 91.5546617487

H7 132.8273921469 111.2944233334 91.9645038618

H8 130.9046764019 111.8914468068 90.3725381300

H9 130.8524067834 110.2193103853 89.8683239345

S10 129.2061044674 111.7565301772 97.3281684143

C11 130.6410589196 112.8753544643 93.1648875935

C12 127.9318385832 115.4274177647 96.2398485163

C13 125.0736685622 111.5652261151 96.8351886938

C14 128.4193431279 108.8432275395 94.6345897041

N15 129.0822450186 113.7985429892 94.8101160762

C16 130.0599169437 113.9326259373 93.8439526408

C17 130.4360135507 115.3290676962 93.6641481049

C18 129.7190123826 116.0186647346 94.5991074377

C19 128.8852480246 115.0749673701 95.2970163010

N20 126.7928340685 113.2552271272 96.3650229006

C21 126.9292152735 114.5821956305 96.7008334535

C22 125.7992170434 115.0218176358 97.4815049794

C23 124.9602976082# 113.9563979373# 97.5889964608#

C24 125.5913929825 112.8520488193 96.9067539328

N25 126.9337511246 110.5466933935 95.6014957653

C26 125.7211504958 110.4779771582 96.2586553907

C27 125.2687991599# 109.1083982111# 96.3358988929#

C28 126.2439490094 108.3439728977 95.7657903599

C29 127.2734276411 109.2420145154 95.3120374467

N30 129.3135832099 111.0798345344 94.1745340999

C31 129.3429457588 109.7058319041 94.0634398196

C32 130.3949236732 109.3011928847 93.1675017486

C33 130.9912234641 110.4336962488 92.6906813817

C34 130.3137293644 111.5380555070 93.3465213680

H35 130.9904895709 110.2678763867 96.5591374831

H36 131.4983006637 111.3585821630 97.8750758444

H37 131.4286467082 113.1127087080 92.4591974404

H38 127.8919059041 116.4681638194 96.5449031793

H39 124.1171566469 111.3830217801 97.3158648906

H40 128.5573437060 107.7788418487 94.4694430417

H41 129.6724492302 117.0913815787 94.7378556575

H42 130.6188119799 108.2742021994 92.9043096611

H43 126.2702879606 107.2681950755 95.6452725538

H44 124.3378962167 108.7905334506 96.7877626211

H45 124.0009803755 113.9025380878 98.0881003397

H46 125.6658039105 116.0277260716 97.8580441051

C47 130.9292997130# 111.2900001360# 96.9415999048#

H48 131.3692001469# 111.9767999306# 96.2091000231#

O49 133.7879001442 111.4337914154 89.5411315553

C50 131.3033704045 115.8641730061 92.5627157101

H51 131.6502248799 116.8690558183 92.8249765262

H52 132.2047448661 115.2511004736 92.4347022461

C53 130.5280519266 115.9201319930 91.2294628263

H54 130.0558590685 114.9506428036 91.0308912749

H55 129.7042408742 116.6400615235 91.3121883695

C56 131.3489981683# 116.2862016062# 89.9720001492#

O57 132.4866738494 116.8462703870 90.1412635278

O58 130.8234046709 115.9930741035 88.8758142501

H59 132.8540380699 111.6885875800 87.4668254990

C60 138.0599985507# 116.3180010825# 94.3590003434#

C61 137.6124144416 114.8858418746 93.9845719854

C62 138.6358000106 114.1262632493 93.1754042473

C63 139.7277952698 113.4681435591 93.6860553248

C64 138.7289635530 113.9896971547 91.7387069579

N65 140.5167081419 112.9669336637 92.6606336328

C66 139.9097215248 113.2472949358 91.4523483637

C67 137.9213216428 114.4103550931 90.6663675041

C68 140.2998100487 112.9311092202 90.1455868383

C69 138.2969838478 114.0908225821 89.3658788076

C70 139.4774774737 113.3611574820 89.1074305328

H71 138.0245505748# 116.9636993594# 93.5429379729#

H72 136.6624827092 114.9292114337 93.4383382866

H73 137.4059092755 114.3262687741 94.9069764683

H74 140.0322157358 113.3387125986 94.7164780205

H75 141.2890862841 112.3314037471 92.7851944807

H76 137.0139445138 114.9766462576 90.8496290802

H77 141.2070836769 112.3651412340 89.9483707018

H78 137.6766485344 114.4144560086 88.5347210629

H79 139.7461863605 113.1239286129 88.0814288833

C80 134.2678970814 124.5985402243 82.5161003550

C81 134.6040727350 123.8647603258 83.8057867392

O82 134.7739018121 124.4265444170 84.8811182968

H83 135.3452097743 126.4051282293 83.0723390085

C84 134.4188080208# 126.1216049815# 82.5638730236#

H85 133.5937649918# 126.6247940081# 83.0715709786#

H86 134.4691309831# 126.4689410175# 81.5301900050#

H87 133.2456008001 124.3126390391 82.2335334329

H88 134.9094181885 124.1757699511 81.7331401610

N89 136.8879431041 118.0799701277 91.2611719729

C90 135.6956766554 117.7374318010 90.7878332501

N91 134.6157749168 117.7205144339 91.5897171991

N92 135.4969641745 117.3819113172 89.4769682643

C93 136.5401238955 117.3185552912 88.5314055062

O94 136.3069399457 116.9978621684 87.3508179250

C95 137.8291287025 117.6531916990 89.0561059264

C96 137.9316780274 118.0284948659 90.3962734926

N97 138.9737626300 117.6234802277 88.2378111737

N98 139.1659244771 118.3416928229 90.9031280496

C99 140.2411792385# 117.3935382833# 88.9400336918#

C100 140.3464053229# 118.4304436585# 90.0548097754#

H101 133.6913347178 117.4076307929 91.2339716439

H102 134.7598617400 117.9753174851 92.5541487036

H103 134.5537650826 117.0889547993 89.1983085170

H104 138.8214776929 117.0542235303 87.4110269082

H105 139.1842255718 118.6781697822 91.8549347522

H106 140.3081955887 116.3835651417 89.3720718405

H107 140.4503947976 119.4321002293 89.6116014023

H108 141.2348092849 118.2373608405 90.6672524365

H109 141.0651216183 117.5252813125 88.2307081250

C110 131.8899992156# 123.9739989567# 86.7559968649#

C111 131.7147392563 122.5117179871 87.2418966466

C112 132.2954588456 122.1137632153 88.6135780116

C113 133.7744279737 121.6885954053 88.6812530762

N114 134.0978809514 120.4733331716 87.9369367493

C115 134.5850016194 120.4749742307 86.6484260259

N116 134.9862889862 121.5232202788 85.9900574233

H117 132.9138441412 124.2115537777 86.4534488951

H118 132.1025901108 121.8303506292 86.4745661267

H119 130.6375914589 122.3052886905 87.2881729982

H120 131.7080495599 121.2670828198 88.9933762786

H121 132.1459360047 122.9318154617 89.3341954353

H122 134.0366351886 121.5078980666 89.7317506166

H123 134.4414433652 122.4859237802 88.3406952783

H124 133.5627407737 119.6508450019 88.1942421543

H125 134.7994865226 122.4008912856 86.4677765667

H126 135.1539286013 119.2619577878 85.1779464493

H127 131.5752869691# 124.6768798889# 87.5273740890#

H128 131.2467799116# 124.0445940080# 85.8788530652#

N129 134.6376202597 119.2313494844 86.0484201659

H130 134.9318596255 118.4384674545 86.6201443284

O131 134.6824200559 122.5573010257 83.6019889709

H132 134.8639471955 122.0617847458 84.4924197755

H133 137.5133210177# 116.7113415412# 95.0982062570#

H134 139.0995272355 116.2876559057 94.7133198231

C135 128.9210047623# 122.2570040139# 90.4810027722#

C136 128.0321117126 121.0383247758 90.7655039523

C137 128.4893063336 120.2120374800 91.9804272345

C138 127.7976445863 118.8435630110 92.0985060759

O139 127.6679606365 118.1993211247 91.0053158272

O140 127.4434688679 118.4348683404 93.2379753740

H141 129.9385990729# 121.9481960300# 90.2378012336#

H142 128.0162496842 120.3971185120 89.8828224825

H143 126.9958737138 121.3695202247 90.9283190487

H144 128.3506358247 120.7621084502 92.9182743465

H145 129.5675390878 120.0085423962 91.8877669689

H146 128.9394990943# 122.9180979983# 91.3473969993#

H147 128.5392543732 122.8296778969 89.6271674692

C148 126.2973792134 115.1937191863 91.9915837883

N149 126.3345243867 115.9010426308 93.1258258150

H150 126.7343694302 116.8768021058 93.1481013099

H151 126.3073412938 115.3792453004 93.9908261594

N152 125.9830647912 113.8493859273 91.9473380488

O153 125.7196181311 113.4226697400 93.2291725192

N154 129.2229623591 118.3522592119 88.2141250260

C155 128.1100709078 118.0003011282 87.3497433696

C156 127.4928972166# 119.2174987488# 86.6662981692#

C157 126.9563099233 117.2023796447 88.0330501741

C158 127.3999669059 115.7977790895 88.4815884137

C159 126.4672177483 115.1290782669 89.5035473611

N160 126.5587042514 115.7742597394 90.8191654754

O161 126.7050808162 119.1589692809 85.7379485122

H162 128.8778033033 118.4930152541 89.1650188474

H163 128.4921419548 117.3928539769 86.5181651739

H164 126.1067978564 117.1242955496 87.3422866409

H165 126.6243684323 117.7918912425 88.8953171932

H166 128.4215162055 115.8241015339 88.8783680987

H167 127.4370239314 115.1385224508 87.6030471895

H168 126.7202952558 114.0711572005 89.6120505990

H169 125.4237772352 115.1762037893 89.1561994598

H170 126.9408220090 116.7619891826 90.8810285537

O171 127.8607959146 120.4087893863 87.1964227299

H172 127.3615673750 121.0701259676 86.6806516452

H173 129.8807697056 117.5706764995 88.2630632814

H174 125.6181753451 112.4591241797 93.1404715767

The structure when a (H^+^,e^-^) has been added to the starting structure

Energies: E= -4728.595586, solv = -0.106253, disp = -180.47 Z_0_ = 898.50

Fe1 128.0413515706 112.2088246427 95.2510071837

C2 132.0911721220 110.6125865705 91.6662528398

C3 131.5268234494 111.0363898107 90.3016195196

C4 132.6118008844# 111.2870028867# 89.2855001769#

O5 132.0864725467 111.6496238646 88.0920967452

H6 132.6229434262 109.6610078607 91.5597667595

H7 132.8433870684 111.3445165194 91.9807409023

H8 130.9299089012 111.9525760376 90.3853377089

H9 130.8421540460 110.2761639963 89.9053244129

S10 129.5728063757 112.1773305327 97.7941593719

C11 130.6532253869 112.9226844100 93.1706181117

C12 127.8528960169 115.4930589940 96.1415315537

C13 125.0845508719 111.5674253388 96.8545177982

C14 128.3763168959 108.8709481294 94.5442238549

N15 129.0609065468 113.8690317989 94.7650461878

C16 130.0626603626 113.9918823394 93.8300107972

C17 130.4477448359 115.3894975467 93.6554586435

C18 129.6958716330 116.0877474152 94.5543909083

C19 128.8361581743 115.1457959606 95.2268629528

N20 126.7376461190 113.3035575692 96.2844501245

C21 126.8609283972 114.6332855662 96.6077005655

C22 125.7575553785 115.0481110990 97.4355061235

C23 124.9602977963# 113.9563978078# 97.5889963867#

C24 125.5783418363 112.8682947486 96.8824712078

N25 126.9291417609 110.5496491571 95.6024640896

C26 125.7250440814 110.4775252107 96.2732951748

C27 125.2687992729# 109.1083982981# 96.3358989031#

C28 126.2232322218 108.3508314271 95.7217283939

C29 127.2466175646 109.2545550476 95.2621155412

N30 129.2750631191 111.1188978266 94.1111382296

C31 129.2982736986 109.7456108999 93.9847503817

C32 130.3769372541 109.3467884992 93.1186703859

C33 130.9978512747 110.4809807986 92.6815198673

C34 130.3061528490 111.5845932992 93.3256682877

H35 130.5529936096 110.4161490128 96.4091721024

H36 131.7035776667 110.9926534692 97.6534165107

H37 131.4656230562 113.1532029457 92.4909749524

H38 127.7956797682 116.5340746804 96.4433031100

H39 124.1455051728 111.3818958148 97.3677803025

H40 128.5119240748 107.8105178053 94.3525903718

H41 129.6436337164 117.1615956035 94.6817300100

H42 130.6062417685 108.3224025912 92.8494890662

H43 126.2373755801 107.2778865490 95.5750759852

H44 124.3475992838 108.7841009721 96.8033807568

H45 124.0312870828 113.8804283579 98.1410079440

H46 125.6151372728 116.0488754003 97.8223626954

C47 130.9292992308# 111.2900003946# 96.9415997398#

H48 131.3692004381# 111.9767997868# 96.2091000631#

O49 133.8052847797 111.1865276471 89.4782959503

C50 131.3243890633 115.9146691598 92.5617361249

H51 131.6686008405 116.9237645910 92.8109152780

H52 132.2259299937 115.3019438362 92.4361397805

C53 130.5376195711 115.9471762535 91.2366370977

H54 130.0624799973 114.9746800108 91.0610373238

H55 129.7145132105 116.6686821402 91.3133194990

C56 131.3489980833# 116.2862015671# 89.9720002395#

O57 132.5008148989 116.8181156593 90.1252594388

O58 130.8018140799 116.0019949268 88.8825080297

H59 132.8400717752 111.8720149853 87.5131285744

C60 138.0599984301# 116.3180009340# 94.3590004450#

C61 137.5541956795 114.8902565382 94.0197299014

C62 138.6140894069 113.9332980054 93.5380263708

C63 139.3493868311 113.0933086354 94.3371575998

C64 139.0943434302 113.7203907287 92.1912016903

N65 140.2725797112 112.3883626494 93.5786955637

C66 140.1212771485 112.7354186148 92.2515303348

C67 138.7371436252 114.2481416943 90.9370287773

C68 140.7884533319 112.2754046297 91.1096813033

C69 139.3901742820 113.7868004060 89.7992173168

C70 140.4080886161 112.8126346224 89.8837252415

H71 138.0245505725# 116.9636992964# 93.5429379333#

H72 136.7452359915 114.9576410531 93.2818611619

H73 137.1003786765 114.4616947376 94.9232411651

H74 139.2986930587 112.9463338308 95.4080373218

H75 140.8558515092 111.6413562997 93.9205437723

H76 137.9613271849 115.0029562188 90.8540095523

H77 141.5694194325 111.5218325699 91.1767393921

H78 139.1079645006 114.1764706609 88.8251686396

H79 140.8987483104 112.4708734981 88.9762197301

C80 134.2761802730 124.5981659442 82.5192198667

C81 134.6143396662 123.8730310573 83.8131307615

O82 134.7640300263 124.4440113490 84.8872620310

H83 135.3442784163 126.4093005650 83.0717527672

C84 134.4188080052# 126.1216049572# 82.5638730406#

H85 133.5937649966# 126.6247940263# 83.0715709684#

H86 134.4691309911# 126.4689410166# 81.5301900051#

H87 133.2563249093 124.3047113173 82.2353257510

H88 134.9224235700 124.1770360791 81.7392616991

N89 136.9011462011 118.0058102033 91.2836127888

C90 135.7095280606 117.6682818932 90.8104536669

N91 134.6270313993 117.6537359845 91.6152281862

N92 135.5086953855 117.3235980162 89.4993532561

C93 136.5468584604 117.2848141214 88.5441890893

O94 136.2976968077 117.0106515693 87.3541624908

C95 137.8364328043 117.5932597551 89.0736126514

C96 137.9437301779 117.9680134021 90.4097249249

N97 138.9778213740 117.6170419989 88.2341146606

N98 139.1775379074 118.3059793503 90.9166338905

C99 140.2411790676# 117.3935382901# 88.9400336946#

C100 140.3464054517# 118.4304436569# 90.0548097687#

H101 133.7042268749 117.3437964930 91.2577309318

H102 134.7781762018 117.8749995848 92.5862427486

H103 134.5614926039 117.0531671790 89.2139510651

H104 138.8374132203 117.0297014328 87.4175573520

H105 139.1772872739 118.7191676348 91.8384293207

H106 140.3208433359 116.3868920259 89.3803916813

H107 140.4243349842 119.4351963209 89.6132294959

H108 141.2469083803 118.2502659778 90.6543164857

H109 141.0672950979 117.5276009541 88.2330246810

C110 131.8899991939# 123.9739989471# 86.7559968860#

C111 131.7237771105 122.5086642559 87.2378899006

C112 132.3015444644 122.1064401197 88.6101929463

C113 133.7850190260 121.6945898889 88.6881085296

N114 134.1322844655 120.4889097083 87.9412398838

C115 134.6270337013 120.4981464326 86.6579990894

N116 134.9998457921 121.5582345770 85.9999177285

H117 132.9124154867 124.2162827085 86.4523325765

H118 132.1197795387 121.8338890099 86.4690362482

H119 130.6478732158 122.2944696401 87.2791579769

H120 131.7178921141 121.2521910313 88.9792695053

H121 132.1401019010 122.9185334125 89.3351683187

H122 134.0369637789 121.5084209469 89.7400269758

H123 134.4477314268 122.5015028828 88.3614310516

H124 133.6476765848 119.6426907648 88.2183837057

H125 134.7900681447 122.4306025454 86.4771790557

H126 135.2400885447 119.3011842097 85.1937821376

H127 131.5752869765# 124.6768798990# 87.5273740827#

H128 131.2467799157# 124.0445940138# 85.8788530625#

N129 134.7175479458 119.2574358022 86.0597288459

H130 135.0264166468 118.4727440550 86.6354867582

O131 134.7183276227 122.5679472534 83.6156659144

H132 134.8954631351 122.0787238442 84.5168266529

H133 137.5133211273# 116.7113417117# 95.0982062474#

H134 139.0970278551 116.2660939896 94.7146949150

C135 128.9210047767# 122.2570039658# 90.4810028211#

C136 128.0192022778 121.0426231638 90.7485920591

C137 128.4548031452 120.1987536525 91.9596137695

C138 127.7403620358 118.8409996857 92.0667315748

O139 127.6014790571 118.2029921501 90.9705766121

O140 127.3760558873 118.4302428541 93.2028731856

H141 129.9385990825# 121.9481960951# 90.2378011542#

H142 128.0052389139 120.4106533450 89.8590994073

H143 126.9845483872 121.3819583384 90.9047648451

H144 128.3204305964 120.7443733063 92.9006279934

H145 129.5298686869 119.9780170149 91.8714735680

H146 128.9394990925# 122.9180979883# 91.3473970070#

H147 128.5463728148 122.8358643338 89.6279703613

C148 126.2961302680 115.1963787452 91.9479780379

N149 126.2902884491 115.9100275339 93.0759622242

H150 126.6812326642 116.8950099768 93.0990691888

H151 126.2701356012 115.3945135675 93.9456013373

N152 126.0164864293 113.8348795221 91.9102231269

O153 125.7459846360 113.4157481310 93.1831328803

N154 129.2117982153 118.3611655383 88.2307625297

C155 128.1249119957 118.0017434401 87.3387679949

C156 127.4928972038# 119.2174987518# 86.6662981674#

C157 126.9740417682 117.1710380629 87.9840828481

C158 127.4424609055 115.7821898084 88.4519371749

C159 126.5050014307 115.1061327808 89.4631413108

N160 126.5634352408 115.7652747343 90.7759877790

O161 126.6949821450 119.1562418455 85.7464896826

H162 128.8431893102 118.4869296618 89.1742188967

H163 128.5373511168 117.4168343946 86.5052106788

H164 126.1500549444 117.0692817554 87.2662700531

H165 126.5975139163 117.7523892083 88.8334604067

H166 128.4566155716 115.8337717324 88.8651851321

H167 127.5086327069 115.1145722609 87.5815394361

H168 126.7724562886 114.0535842751 89.5857561609

H169 125.4664081455 115.1363652717 89.1007528132

H170 126.9238240766 116.7687162477 90.8376268700

O171 127.8620970091 120.4099296478 87.1920198837

H172 127.3559967828 121.0688307541 86.6797102906

H173 129.8817494000 117.5901317604 88.2861729122

H174 125.6709204287 112.4474185836 93.1117672774

H175 129.0443913736 111.1037124208 98.4200100919

The structure in Figure 2 when O_2_ has been added

Energies: E= -4878.906191, solv = -0.108922, disp = -185.54 Z_0_ = 902.87

Fe1 128.0238285627 112.2300889861 95.2366882220

C2 132.0941532268 110.6135881226 91.6782838774

C3 131.5276750545 111.0356727351 90.3108176576

C4 132.6118008337# 111.2870028928# 89.2855003072#

O5 132.0785200751 111.6115441410 88.0859508931

H6 132.6215335936 109.6595054119 91.5713730989

H7 132.8531092385 111.3422959989 91.9841399790

H8 130.9311387289 111.9523380016 90.3941599770

H9 130.8414352934 110.2755393927 89.9176337405

S10 129.4023687744 112.1312901177 97.5051441871

C11 130.6909665341 112.9237349221 93.2161929800

C12 127.8504802282 115.4998409955 96.1488549898

C13 125.0987029852 111.5615399370 96.8920630030

C14 128.4297642819 108.8761844052 94.6330131244

N15 129.1080059954 113.8670918431 94.8242366394

C16 130.0967836873 113.9888663581 93.8755750265

C17 130.4549243419 115.3879750880 93.6707397545

C18 129.6910568039 116.0892928884 94.5601218116

C19 128.8460346077 115.1485126062 95.2501192224

N20 126.7712381187 113.2931432214 96.3508557109

C21 126.8716991043 114.6356792404 96.6293939888

C22 125.7493848720 115.0544007296 97.4250495283

C23 124.9602978608# 113.9563977726# 97.5889963432#

C24 125.5994434116 112.8590828601 96.9235359189

N25 126.9818450670 110.5417249051 95.7126829152

C26 125.7488721354 110.4724964761 96.3251793274

C27 125.2687992848# 109.1083983215# 96.3358988996#

C28 126.2425854583 108.3542647074 95.7470065268

C29 127.2943766545 109.2556440852 95.3441507513

N30 129.3437731045 111.1225886991 94.2039658295

C31 129.3510497073 109.7518001427 94.0713531904

C32 130.4062115552 109.3519903733 93.1757698196

C33 131.0166835600 110.4849915829 92.7167667637

C34 130.3484694854 111.5877365229 93.3852459431

H35 130.6935261780 110.3496497404 96.4455048752

H36 131.6201147826 111.1373143708 97.7734891795

H37 131.4852430355 113.1558541722 92.5167353181

H38 127.7684020320 116.5475230397 96.4189242704

H39 124.1386421916 111.3868738478 97.3677263952

H40 128.5576619530 107.8183662253 94.4245675378

H41 129.6242636465 117.1640021199 94.6732899016

H42 130.6213064313 108.3267336992 92.8987624747

H43 126.2496158385 107.2859361036 95.5701325441

H44 124.3234128809 108.7877785445 96.7539831965

H45 124.0199670711 113.8840567014 98.1217408583

H46 125.5877950332 116.0628603620 97.7830801486

C47 130.9292991758# 111.2900003722# 96.9415996569#

H48 131.3692004749# 111.9767998031# 96.2091001004#

O49 133.8063575219 111.2176377420 89.4833903219

C50 131.3303599470 115.9047707031 92.5694444725

H51 131.6641794944 116.9202115197 92.8063511498

H52 132.2385434330 115.2989071646 92.4591802105

C53 130.5473647510 115.9179310959 91.2392644498

H54 130.0928015823 114.9355327302 91.0633216136

H55 129.7137725263 116.6256563280 91.3181129609

C56 131.3489979861# 116.2862016940# 89.9720001428#

O57 132.4882868412 116.8403448993 90.1279210349

O58 130.8015234437 115.9973990794 88.8836716506

H59 132.8260167441 111.8414247240 87.5017863014

C60 138.0599986268# 116.3180010186# 94.3590003656#

C61 137.5427898133 114.8941677428 94.0212760665

C62 138.5939926242 113.9266650319 93.5437136293

C63 139.3111205926 113.0719743570 94.3441056290

C64 139.0743214208 113.7073568115 92.1987620343

N65 140.2217724224 112.3499481464 93.5870505862

C66 140.0864526742 112.7077097151 92.2613072918

C67 138.7307804518 114.2451125239 90.9449216586

C68 140.7528209857 112.2417996359 91.1215880576

C69 139.3838507379 113.7789212849 89.8090615149

C70 140.3864736165 112.7894534071 89.8964979271

H71 138.0245505544# 116.9636993643# 93.5429378269#

H72 136.7372874881 114.9692436577 93.2802792568

H73 137.0818514200 114.4696249828 94.9232885232

H74 139.2504544834 112.9195717688 95.4137158804

H75 140.8137129462 111.6125799274 93.9344914956

H76 137.9669986603 115.0117676773 90.8598159237

H77 141.5223022934 111.4765049124 91.1897915753

H78 139.1117991590 114.1775191721 88.8359368490

H79 140.8774342713 112.4453281397 88.9900463040

C80 134.2747272675 124.5985290478 82.5200165843

C81 134.6099836855 123.8745055443 83.8156747893

O82 134.7612643885 124.4485484451 84.8882952126

H83 135.3444011356 126.4083959595 83.0721735554

C84 134.4188079972# 126.1216049381# 82.5638730440#

H85 133.5937649994# 126.6247940326# 83.0715709664#

H86 134.4691309958# 126.4689410230# 81.5301900076#

H87 133.2550044341 124.3062520424 82.2343976331

H88 134.9218665946 124.1760595529 81.7415003693

N89 136.9063531933 117.9963926560 91.2940999199

C90 135.7146599460 117.6633679248 90.8215250050

N91 134.6324478593 117.6440889020 91.6305499579

N92 135.5084962843 117.3269672848 89.5103113847

C93 136.5446471029 117.2889364363 88.5511372110

O94 136.2848286187 117.0195855905 87.3611616074

C95 137.8339500216 117.5913746053 89.0787489811

C96 137.9464928370 117.9600738801 90.4136983912

N97 138.9787156245 117.6220951883 88.2304881632

N98 139.1823929658 118.2980485249 90.9208263386

C99 140.2411789656# 117.3935381245# 88.9400338518#

C100 140.3464055123# 118.4304438022# 90.0548097598#

H101 133.7059751807 117.3520620467 91.2729747133

H102 134.7841283217 117.8824100432 92.5971101812

H103 134.5615271491 117.0585430739 89.2248237935

H104 138.8368278642 117.0160412358 87.4275082693

H105 139.1817378793 118.7118639881 91.8415042134

H106 140.3201402535 116.3879255291 89.3829472197

H107 140.4202452428 119.4359537529 89.6135589860

H108 141.2504326125 118.2531249656 90.6503956339

H109 141.0687233716 117.5266400840 88.2338029489

C110 131.8899991931# 123.9739989559# 86.7559968976#

C111 131.7257408639 122.5084573688 87.2375269986

C112 132.3035162282 122.1068331160 88.6099779529

C113 133.7882100463 121.7002809282 88.6897605158

N114 134.1405951368 120.4955791292 87.9446548799

C115 134.6354666468 120.5032954739 86.6613600456

N116 135.0005661730 121.5658349839 86.0017523227

H117 132.9122508843 124.2167774574 86.4519824075

H118 132.1234180596 121.8344671546 86.4688715650

H119 130.6500772575 122.2924822573 87.2782967054

H120 131.7227792999 121.2500707246 88.9774867190

H121 132.1388747793 122.9179933202 89.3355403945

H122 134.0399142022 121.5170972630 89.7422984827

H123 134.4471748290 122.5102070978 88.3628543503

H124 133.6704047092 119.6442988909 88.2304883076

H125 134.7894516408 122.4368909826 86.4806603136

H126 135.2631507252 119.3076384018 85.2041482660

H127 131.5752869775# 124.6768799058# 87.5273740770#

H128 131.2467799125# 124.0445940089# 85.8788530644#

N129 134.7356084025 119.2624565588 86.0670493193

H130 135.0448004442 118.4761824755 86.6437855356

O131 134.7085545177 122.5692184053 83.6214208547

H132 134.8887887360 122.0795223448 84.5244583741

H133 137.5133210258# 116.7113415392# 95.0982062642#

H134 139.0971906296 116.2610161071 94.7130082527

C135 128.9210047633# 122.2570040318# 90.4810027650#

C136 128.0343362643 121.0319522886 90.7537377463

C137 128.4885535098 120.2005969914 91.9701396526

C138 127.7890262523 118.8369151546 92.0649440121

O139 127.8899003344 118.0890389066 91.0400990859

O140 127.1780222331 118.5370561357 93.1328940617

H141 129.9385990799# 121.9481960144# 90.2378012135#

H142 128.0318551054 120.3967600309 89.8655058500

H143 126.9951640414 121.3559096871 90.9115854889

H144 128.3311280221 120.7506041251 92.9046826285

H145 129.5662716678 120.0019815720 91.8785884871

H146 128.9394990668# 122.9180980050# 91.3473969947#

H147 128.5400816670 122.8301764283 89.6273456298

C148 125.7307756917 115.5546743769 91.6991679402

N149 125.6872276410 116.3397421861 92.7697628518

H150 126.3224018758 117.1950917587 92.9122549840

H151 124.9973456229 116.1065990689 93.4707144807

N152 124.6772803085 114.6130119423 91.5990035183

O153 125.0878520759 113.3447166260 91.3964137724

N154 129.2710341357 118.3716996669 88.1688897741

C155 128.1505020567 118.0073022840 87.3202687052

C156 127.4928973031# 119.2174986706# 86.6662982445#

C157 127.0233088180 117.1773174542 88.0079804042

C158 127.5052825719 115.7780702441 88.4213476243

C159 126.5924038852 115.0567364606 89.4187830265

N160 126.6204357587 115.7251850598 90.7360930472

O161 126.7060451935 119.1559275218 85.7375664201

H162 128.9429647012 118.5013757651 89.1270057761

H163 128.5310074478 117.4196102642 86.4741062716

H164 126.1630768247 117.0960278112 87.3308740645

H165 126.6957168000 117.7415839181 88.8893648273

H166 128.5253972978 115.8249920000 88.8225265372

H167 127.5689546554 115.1399693446 87.5290998745

H168 126.9305179354 114.0210222896 89.5330181418

H169 125.5556559989 115.0225859682 89.0604139452

H170 127.2180216060 116.5912303356 90.8518875241

O171 127.8200206972 120.4067813543 87.2274926291

H172 127.3008978411 121.0646629823 86.7271990251

H173 129.9376990969 117.5968028849 88.2104162898

H174 126.0319824161 113.2049849230 91.7120269815

H175 128.7949943179 111.0609492666 98.0608606569

O176 126.9018361900 112.5039238346 93.7803218277

O177 127.4342566784 112.8153713288 92.6453921380

The O--H TS structure in Figure 3.

Energies: E= -4878,887407 solv = -0.116773, disp = -186.96 Z_0_ = 899.85 i1923

Fe1 127.9795660000 112.2064120000 95.1432970000

C2 132.0875890000 110.5820560000 91.6597270000

C3 131.5250680000 111.0136470000 90.2949290000

C4 132.6118010000 111.2870030000 89.2855000000

O5 132.0956950000 111.5615060000 88.0648140000

H6 132.6093760000 109.6247550000 91.5506150000

H7 132.8478320000 111.3063700000 91.9719310000

H8 130.9216840000 111.9250190000 90.3890390000

H9 130.8447760000 110.2553480000 89.8891630000

S10 129.2891420000 112.0075150000 97.3233690000

C11 130.7052830000 112.8928680000 93.2154800000

C12 127.7266430000 115.5183710000 95.9540240000

C13 125.1316790000 111.5438360000 96.9385980000

C14 128.3264520000 108.8685090000 94.4796460000

N15 129.0457440000 113.8571540000 94.7281800000

C16 130.0824820000 113.9655910000 93.8364470000

C17 130.4425900000 115.3624350000 93.6246690000

C18 129.6150680000 116.0811390000 94.4336150000

C19 128.7489370000 115.1450820000 95.1000060000

N20 126.7253290000 113.2945790000 96.2711460000

C21 126.7872210000 114.6488180000 96.4905780000

C22 125.6936860000 115.0710290000 97.3234860000

C23 124.9602980000 113.9563980000 97.5889960000

C24 125.6002350000 112.8527780000 96.9255620000

N25 126.9446270000 110.5335920000 95.6347710000

C26 125.7546190000 110.4663200000 96.3199620000

C27 125.2687990000 109.1083980000 96.3358990000

C28 126.1851450000 108.3527120000 95.6693670000

C29 127.2207460000 109.2528300000 95.2283810000

N30 129.2959190000 111.1003160000 94.1242520000

C31 129.2712370000 109.7358490000 93.9517200000

C32 130.3362480000 109.3296290000 93.0735740000

C33 130.9948790000 110.4568080000 92.6770050000

C34 130.3348330000 111.5603730000 93.3508790000

H35 130.8281320000 110.3080940000 96.4786790000

H36 131.5495790000 111.2430840000 97.8388630000

H37 131.5354420000 113.1181090000 92.5557460000

H38 127.6182430000 116.5761110000 96.1709940000

H39 124.2061210000 111.3504700000 97.4726920000

H40 128.4279640000 107.8139700000 94.2408890000

H41 129.5105950000 117.1569950000 94.4980260000

H42 130.5271400000 108.3073820000 92.7680850000

H43 126.1664210000 107.2882320000 95.4710580000

H44 124.3466420000 108.7901290000 96.8064550000

H45 124.0530680000 113.8743700000 98.1755530000

H46 125.5185700000 116.0902610000 97.6434970000

C47 130.9292990000 111.2900000000 96.9416000000

H48 131.3692000000 111.9768000000 96.2091000000

O49 133.8047110000 111.2786750000 89.5054660000

C50 131.3421880000 115.8735460000 92.5452970000

H51 131.7019660000 116.8760650000 92.8013940000

H52 132.2316730000 115.2439820000 92.4180410000

C53 130.5481860000 115.9305070000 91.2259210000

H54 130.0626900000 114.9653670000 91.0413740000

H55 129.7342030000 116.6593960000 91.3204810000

C56 131.3489980000 116.2862020000 89.9720000000

O57 132.5128360000 116.8148950000 90.1322040000

O58 130.8214000000 116.0238260000 88.8724590000

H59 132.8502380000 111.8056760000 87.4962640000

C60 138.0599990000 116.3180010000 94.3590000000

C61 137.6021700000 114.8796990000 94.0232260000

C62 138.6214550000 114.0668820000 93.2668940000

C63 139.6462740000 113.3439190000 93.8260900000

C64 138.7535650000 113.9039600000 91.8372400000

N65 140.4223030000 112.7619570000 92.8358420000

C66 139.8889440000 113.0775340000 91.6028790000

C67 138.0111740000 114.3551020000 90.7317310000

C68 140.2964280000 112.7095840000 90.3156470000

C69 138.4079890000 113.9911950000 89.4497160000

C70 139.5429400000 113.1783270000 89.2440640000

H71 138.0245510000 116.9636990000 93.5429380000

H72 136.6592070000 114.9119370000 93.4632890000

H73 137.3739760000 114.3636760000 94.9655360000

H74 139.8938980000 113.1973010000 94.8694580000

H75 141.1881630000 112.1270080000 92.9945460000

H76 137.1307040000 114.9729200000 90.8771990000

H77 141.1653000000 112.0746650000 90.1581470000

H78 137.8315920000 114.3346260000 88.5950820000

H79 139.8266130000 112.9032510000 88.2314510000

C80 134.2774530000 124.5974660000 82.5126520000

C81 134.6791870000 123.8690130000 83.7842970000

O82 134.8228980000 124.4299370000 84.8641340000

H83 135.3452940000 126.4094070000 83.0703350000

C84 134.4188080000 126.1216050000 82.5638730000

H85 133.5937650000 126.6247940000 83.0715710000

H86 134.4691310000 126.4689410000 81.5301900000

H87 133.2451160000 124.3037300000 82.2779080000

H88 134.8863000000 124.1832630000 81.7000330000

N89 136.8880870000 118.0545940000 91.2930760000

C90 135.6890000000 117.7108290000 90.8167740000

N91 134.6068780000 117.6949220000 91.6074010000

N92 135.4873450000 117.3560860000 89.5056240000

C93 136.5224960000 117.3080790000 88.5601960000

O94 136.3017770000 117.0141490000 87.3742910000

C95 137.8215920000 117.6216000000 89.0918720000

C96 137.9332980000 118.0013660000 90.4364800000

N97 138.9492350000 117.5850630000 88.2724690000

N98 139.1768200000 118.3124640000 90.9258520000

C99 140.2411790000 117.3935380000 88.9400340000

C100 140.3464060000 118.4304440000 90.0548100000

H101 133.6797050000 117.3681320000 91.2418180000

H102 134.7448940000 117.9330490000 92.5774860000

H103 134.5330530000 117.0831010000 89.2313030000

H104 138.7892790000 117.0756740000 87.4097480000

H105 139.1985300000 118.7114310000 91.8538410000

H106 140.3350120000 116.3811170000 89.3617590000

H107 140.4211500000 119.4361540000 89.6164630000

H108 141.2455260000 118.2485020000 90.6533450000

H109 141.0411270000 117.5482240000 88.2091180000

C110 131.8899990000 123.9739990000 86.7559970000

C111 131.7443590000 122.5155680000 87.2523880000

C112 132.3858050000 122.1525050000 88.6040030000

C113 133.8872710000 121.8141450000 88.6066010000

N114 134.2287080000 120.6010320000 87.8664250000

C115 134.7680090000 120.5882290000 86.6000100000

N116 135.2523300000 121.6094410000 85.9567560000

H117 132.9108890000 124.2220800000 86.4486380000

H118 132.1201190000 121.8324900000 86.4795540000

H119 130.6733020000 122.2920870000 87.3372860000

H120 131.8563230000 121.2800200000 89.0111020000

H121 132.2242840000 122.9670310000 89.3255380000

H122 134.2156650000 121.6777340000 89.6456220000

H123 134.4808470000 122.6412010000 88.2079040000

H124 133.6689360000 119.7880370000 88.0994850000

H125 135.0974860000 122.5019050000 86.4181530000

H126 135.3316520000 119.3466690000 85.1493730000

H127 131.5752870000 124.6768800000 87.5273740000

H128 131.2467800000 124.0445940000 85.8788530000

N129 134.7747460000 119.3442810000 85.9946170000

H130 134.9944930000 118.5382250000 86.5777890000

O131 134.8471570000 122.5727120000 83.5671190000

H132 135.0676180000 122.0941640000 84.4622500000

H133 137.5133210000 116.7113420000 95.0982060000

H134 139.1000300000 116.2884770000 94.7118260000

C135 128.9210050000 122.2570040000 90.4810030000

C136 128.0534360000 121.0311540000 90.7772200000

C137 128.5564090000 120.2271050000 91.9883330000

C138 127.8742390000 118.8575880000 92.1065150000

O139 127.9316880000 118.1278070000 91.0661760000

O140 127.3194180000 118.5458480000 93.1961330000

H141 129.9385990000 121.9481960000 90.2378010000

H142 128.0371600000 120.3855250000 89.8974710000

H143 127.0147310000 121.3438430000 90.9588830000

H144 128.4172800000 120.7864150000 92.9206490000

H145 129.6345600000 120.0450720000 91.8669860000

H146 128.9394990000 122.9180980000 91.3473970000

H147 128.5319220000 122.8253780000 89.6273610000

C148 125.7496380000 115.5400790000 91.7901090000

N149 125.6777750000 116.3744290000 92.8282300000

H150 126.3305220000 117.1922510000 92.9608140000

H151 125.0956500000 116.0741890000 93.5973840000

N152 124.7721880000 114.5306740000 91.7583460000

O153 125.2414080000 113.3396380000 91.4417410000

N154 129.2588590000 118.3833620000 88.1835840000

C155 128.1321990000 118.0109420000 87.3440180000

C156 127.4928970000 119.2174990000 86.6662980000

C157 127.0029780000 117.2045150000 88.0582670000

C158 127.4777670000 115.8025180000 88.4755600000

C159 126.5810740000 115.0795380000 89.4890690000

N160 126.6158980000 115.7439930000 90.8041020000

O161 126.7727520000 119.1547320000 85.6862830000

H162 128.9354180000 118.5176090000 89.1433390000

H163 128.5023140000 117.4011810000 86.5094560000

H164 126.1326970000 117.1251450000 87.3931470000

H165 126.6951210000 117.7801170000 88.9387780000

H166 128.5035010000 115.8471650000 88.8598510000

H167 127.5258360000 115.1622260000 87.5835040000

H168 126.9228710000 114.0456420000 89.5942890000

H169 125.5411570000 115.0377830000 89.1395720000

H170 127.1974600000 116.6110650000 90.9031030000

O171 127.7476670000 120.4050070000 87.2691150000

H172 127.2381970000 121.0562200000 86.7507950000

H173 129.9235240000 117.6078540000 88.2246120000

H174 126.3537290000 113.0932550000 91.9296930000

H175 128.7110830000 110.8970150000 97.8271000000

O176 126.8258570000 112.4031550000 93.7141750000

O177 127.3382760000 112.8380590000 92.5481210000

The H_2_O_2_ structure in Figure 4

Energies: E= -4878.892934, solv = -0.104808, disp = -190.21 Z_0_ = 902.02

Fe1 128.0576252473 112.2028946257 95.2548495303

C2 132.1380193782 110.5839240410 91.6868522039

C3 131.5534092273 111.0187147213 90.3306352461

C4 132.6118008256# 111.2870028893# 89.2855003214#

O5 132.0524910710 111.5207231790 88.0743975259

H6 132.6622995545 109.6293830224 91.5646549557

H7 132.9009613785 111.3097975215 91.9900950114

H8 130.9585960619 111.9346392022 90.4379097142

H9 130.8569749950 110.2657105882 89.9432683384

S10 129.3964193963 112.1577636894 97.4727177045

C11 130.7649415218 112.8950041308 93.2706176281

C12 127.7766573719 115.5286733121 96.0153025152

C13 125.1334854541 111.5541492664 96.9158395249

C14 128.4001462927 108.8662690909 94.5688622237

N15 129.0853639780 113.8797641905 94.7592586124

C16 130.1421824485 113.9834631829 93.8797363452

C17 130.4983573752 115.3850777408 93.6659221690

C18 129.6819400139 116.1019673649 94.4912237250

C19 128.8062017362 115.1681128296 95.1549952709

N20 126.7245864212 113.3174315598 96.2582139696

C21 126.8048768584 114.6650323277 96.5193460440

C22 125.7030879885 115.0746992506 97.3558717296

C23 124.9602978958# 113.9563977543# 97.5889963326#

C24 125.6003599841 112.8660235371 96.9060973927

N25 126.9838956551 110.5291364026 95.6864132037

C26 125.7715996377 110.4664615386 96.3293054976

C27 125.2687993062# 109.1083983396# 96.3358989001#

C28 126.2111753101 108.3495553174 95.7057968264

C29 127.2712793061 109.2477870802 95.2950257657

N30 129.3805325099 111.0908364045 94.2019619538

C31 129.3547284345 109.7277293590 94.0330739733

C32 130.4144978913 109.3203966699 93.1387939291

C33 131.0640692009 110.4491157357 92.7283166056

C34 130.4082143995 111.5526315416 93.4201533459

H35 130.6883060836 110.3499540508 96.4481713515

H36 131.6108994923 111.1435609531 97.7821722934

H37 131.5898535225 113.1168150611 92.6022196570

H38 127.6886909915 116.5812551594 96.2676371867

H39 124.1971685542 111.3665950391 97.4338900026

H40 128.5042874344 107.8099439360 94.3363585582

H41 129.5920566894 117.1787832309 94.5614577160

H42 130.6022677051 108.2994721957 92.8269582258

H43 126.1956123350 107.2842597074 95.5098277162

H44 124.3291037468 108.7950252214 96.7729425396

H45 124.0505206801 113.8689054806 98.1708194503

H46 125.5313254136 116.0849553834 97.7054148665

C47 130.9292990902# 111.2900004045# 96.9415996221#

H48 131.3692005153# 111.9767997825# 96.2091001053#

O49 133.8109854787 111.3069649517 89.4696527508

C50 131.3772875392 115.8986226478 92.5667000474

H51 131.7175576245 116.9131709433 92.8016679878

H52 132.2787143297 115.2850542177 92.4431892409

C53 130.5767712508 115.9173667829 91.2487739039

H54 130.1272951974 114.9322362423 91.0737298715

H55 129.7351281593 116.6154284297 91.3427553092

C56 131.3489979664# 116.2862017755# 89.9720001211#

O57 132.4979085510 116.8523597968 90.1089049212

O58 130.7976171335 115.9986524173 88.8907082743

H59 132.7851209750 111.7630300307 87.4768042073

C60 138.0599985817# 116.3180010600# 94.3590002337#

C61 137.5719562978 114.8843393177 94.0234792081

C62 138.6463428967 113.9552299622 93.5216032945

C63 139.4018883445 113.1161084274 94.3032889863

C64 139.1272560951 113.7787822759 92.1710426924

N65 140.3365007256 112.4459503581 93.5293167384

C66 140.1815944838 112.8227932070 92.2110939445

C67 138.7527504812 114.3179153436 90.9270037222

C68 140.8641184771 112.4107704761 91.0601570660

C69 139.4215692227 113.9083137344 89.7783410724

C70 140.4708791296 112.9660403336 89.8467883767

H71 138.0245505671# 116.9636993498# 93.5429378540#

H72 136.7497730765 114.9375483979 93.2987557287

H73 137.1409493279 114.4441138121 94.9320332480

H74 139.3523863727 112.9416826585 95.3700003538

H75 140.9583224733 111.7258648139 93.8608705839

H76 137.9404090075 115.0351418880 90.8619807427

H77 141.6664508425 111.6787698825 91.1108225200

H78 139.1261939921 114.3122641241 88.8139102463

H79 140.9745667142 112.6628302600 88.9329866741

C80 134.2716311875 124.5989555253 82.5202185970

C81 134.5990506893 123.8720130170 83.8154189285

O82 134.7693670312 124.4388043061 84.8881948526

H83 135.3447237264 126.4077859109 83.0719223730

C84 134.4188080032# 126.1216049473# 82.5638730517#

H85 133.5937649983# 126.6247940365# 83.0715709608#

H86 134.4691309913# 126.4689410144# 81.5301900044#

H87 133.2529215959 124.3082684012 82.2297632196

H88 134.9212211142 124.1745633353 81.7446968606

N89 136.9154408017 117.9643647857 91.2892953912

C90 135.7160495911 117.6265275553 90.7994111423

N91 134.6426302969 117.5679491497 91.5939049169

N92 135.5061255117 117.3317846027 89.4767487123

C93 136.5208440242 117.3124456371 88.5049661525

O94 136.2432750730 117.1155645600 87.3275843035

C95 137.8840977218 117.5244109872 89.0544337806

C96 137.9577927856 117.9296551464 90.4437407835

N97 138.9374903806 117.3048372893 88.2906330934

N98 139.1782093227 118.2774364948 90.9290076636

C99 140.2411789559# 117.3935380507# 88.9400339271#

C100 140.3464054732# 118.4304438688# 90.0548097238#

H101 133.7036983283 117.2907421649 91.2102619239

H102 134.7772654459 117.7906357840 92.5687151071

H103 134.5446460076 117.0980794384 89.1956632443

H104 126.1280358585 112.9308317940 93.5031090730

H105 139.1966890067 118.6287084671 91.8772409541

H106 140.4768936720 116.3995107244 89.3504273783

H107 140.3797670807 119.4480250466 89.6368535939

H108 141.2533729609 118.2780078207 90.6487669194

H109 140.9941499836 117.6059371031 88.1715829879

C110 131.8899991983# 123.9739989614# 86.7559968971#

C111 131.6951817367 122.5156240700 87.2458385754

C112 132.2583182766 122.1155502549 88.6237614197

C113 133.7302301679 121.6725259708 88.7035063598

N114 134.0436155408 120.4628094540 87.9444937087

C115 134.5202339496 120.4881961500 86.6501570847

N116 134.9474707717 121.5386965595 86.0148263406

H117 132.9162349909 124.2029046498 86.4560832501

H118 132.0791464868 121.8267526182 86.4835647576

H119 130.6151193285 122.3236040424 87.2845032736

H120 131.6570059349 121.2765611034 88.9990210091

H121 132.1122292567 122.9367291818 89.3413739623

H122 133.9782159713 121.4739462490 89.7542417379

H123 134.4114500849 122.4651079988 88.3806244428

H124 133.4697411807 119.6561884133 88.1697562991

H125 134.7863359761 122.4129682985 86.5073763302

H126 135.0488842255 119.2906343429 85.1498824080

H127 131.5752869790# 124.6768799072# 87.5273740764#

H128 131.2467799110# 124.0445940053# 85.8788530651#

N129 134.5315009055 119.2589880903 86.0197689234

H130 134.8090946192 118.4564401679 86.5822534013

O131 134.6711031335 122.5630101711 83.6202839405

H132 134.8432027104 122.0750683794 84.5169133350

H133 137.5133210567# 116.7113414979# 95.0982063089#

H134 139.0974752862 116.2751061409 94.7152325589

C135 128.9210047108# 122.2570040226# 90.4810027230#

C136 128.0440270606 121.0342767653 90.7713009931

C137 128.5354999206 120.2165512836 91.9776839655

C138 127.8334394331 118.8611612923 92.1183566889

O139 127.7213383485 118.1810245152 91.0484893300

O140 127.4369479009 118.4991859388 93.2637757675

H141 129.9385990941# 121.9481960232# 90.2378012769#

H142 128.0216171918 120.3938954072 89.8880537365

H143 127.0079787875 121.3556849918 90.9530060327

H144 128.4248330700 120.7749398820 92.9140566716

H145 129.6087472682 120.0069602580 91.8520154060

H146 128.9394990927# 122.9180980160# 91.3473969858#

H147 128.5351872025 122.8268767247 89.6272923405

C148 125.8849718838 115.5020176420 91.9882414309

N149 126.1146026900 116.1701982736 93.1113504410

H150 126.6638743957 117.0895812982 93.1587987277

H151 125.8258975596 115.7244940221 93.9736385108

N152 125.1751052364 114.2540453303 92.2344060920

O153 124.5145497545 113.8382572163 91.2801780065

N154 129.1730248675 118.3217932463 88.2487776137

C155 128.0606404831 117.9923338460 87.3758417948

C156 127.4928973769# 119.2174985849# 86.6662983015#

C157 126.8679967195 117.2535223097 88.0557712548

C158 127.2403323099 115.8401821946 88.5354290781

C159 126.2302158064 115.2244203450 89.5143995650

N160 126.2813803370 115.9307383548 90.8089237379

O161 126.7430919180 119.1717686543 85.7066600173

H162 128.8314647530 118.4547854510 89.2015462808

H163 128.4286364097 117.3568523755 86.5593398308

H164 126.0255441913 117.1985803121 87.3543406553

H165 126.5463233278 117.8700932776 88.9038270933

H166 128.2428773809 115.8382806926 88.9786747201

H167 127.2968464258 115.1698345216 87.6670016758

H168 126.4560303755 114.1661182104 89.6782492344

H169 125.2130938356 115.2796000546 89.1103018930

H170 126.8534750572 116.8213068916 90.8613168070

O171 127.8550520184 120.4033517797 87.2141043602

H172 127.3869874302 121.0717208503 86.6785643690

H173 129.8276663593 117.5374581989 88.2870148789

H174 128.3128646965 113.3122627124 92.7158447567

H175 128.8080904463 111.1139727605 98.0972456822

O176 126.8948353428 112.3097267433 93.4699410757

O177 127.5855288700 112.8141320092 92.2907775620

The O-O TS structure in Figure 5

Energies: E= -4878.893774, solv = -0.110671, disp = -186.51 Z_0_ = 899.51. i59

Fe1 128.0063141970 112.1909672064 95.1922874174

C2 132.0887271955 110.5409832643 91.6580855389

C3 131.5242598065 110.9715006172 90.2913448734

C4 132.6118007896# 111.2870028801# 89.2855003734#

O5 132.0820543207 111.6226695169 88.0877893734

H6 132.6161874906 109.5875057892 91.5462837899

H7 132.8487280566 111.2675951872 91.9671386047

H8 130.8904175234 111.8616493554 90.3850748569

H9 130.8712197982 110.1959013433 89.8731773801

S10 129.3967242740 112.1331570284 97.4975091461

C11 130.6849848035 112.8557805981 93.2036824569

C12 127.8247338432 115.4930572362 96.0840448024

C13 125.1042106056 111.5568367983 96.8855394191

C14 128.4372893413 108.8311777741 94.6584390209

N15 129.0943226155 113.8238269452 94.8070686251

C16 130.0957881161 113.9276365119 93.8626553709

C17 130.4588648537 115.3227679186 93.6477859417

C18 129.6873455183 116.0411301859 94.5176065191

C19 128.8327932647 115.1170413142 95.2090607918

N20 126.7207466684 113.3037125583 96.2585193649

C21 126.8337044231 114.6413215429 96.5595298344

C22 125.7376733273 115.0570000914 97.3965562944

C23 124.9602979652# 113.9563977207# 97.5889962701#

C24 125.5802377800 112.8640209011 96.8873075420

N25 127.0179401134 110.5135606023 95.7614343363

C26 125.7691498077 110.4639162431 96.3414140659

C27 125.2687993376# 109.1083983812# 96.3358988931#

C28 126.2395526783 108.3409978359 95.7593490181

C29 127.3123203220 109.2274363937 95.3791169905

N30 129.3567296270 111.0577453187 94.2016686839

C31 129.3598351025 109.6912006446 94.0753304373

C32 130.4069222792 109.2799553018 93.1681560203

C33 131.0147846529 110.4086790860 92.7004934869

C34 130.3486099264 111.5160011300 93.3763771392

H35 130.6945454913 110.3501211361 96.4441504374

H36 131.6153783931 111.1392494019 97.7772416273

H37 131.4780583697 113.0883442203 92.5024530712

H38 127.7588221352 116.5432030598 96.3495399198

H39 124.1506471943 111.3731438371 97.3713044512

H40 128.5454611759 107.7704289788 94.4528028522

H41 129.6185797796 117.1178073675 94.6059192059

H42 130.6173114993 108.2528003110 92.8949691481

H43 126.2298924321 107.2745304562 95.5718316618

H44 124.3096362135 108.8010655135 96.7321698979

H45 124.0445305820 113.8758852539 98.1617445840

H46 125.5934503785 116.0614657442 97.7729045432

C47 130.9292990479# 111.2900004049# 96.9415995858#

H48 131.3692005315# 111.9767997871# 96.2091001191#

O49 133.8056024471 111.2567867100 89.4974753910

C50 131.3358252409 115.8373257936 92.5492355953

H51 131.6849814612 116.8455498429 92.7938397281

H52 132.2336287759 115.2197611451 92.4231817723

C53 130.5460874517 115.8796940919 91.2253862791

H54 130.1017746419 114.8980152163 91.0192856416

H55 129.7043414378 116.5766193627 91.3221706907

C56 131.3489978879# 116.2862018282# 89.9720001115#

O57 132.4718397735 116.8704960820 90.1512702442

O58 130.8228155745 115.9962109925 88.8753649984

H59 132.8284111118 111.8901446776 87.5182757250

C60 138.0599985834# 116.3180010521# 94.3590002298#

C61 137.5351398283 114.8954101950 94.0247606746

C62 138.5738703292 113.9198779329 93.5370226497

C63 139.2593328447 113.0273345912 94.3237467488

C64 139.0705790299 113.7278882892 92.1937451999

N65 140.1645809034 112.3059796046 93.5599353916

C66 140.0596497592 112.7056432296 92.2427661510

C67 138.7603164646 114.3093818796 90.9512396680

C68 140.7355469115 112.2616529477 91.0997107817

C69 139.4248868388 113.8710715454 89.8110719047

C70 140.4044577121 112.8573846658 89.8866880771

H71 138.0245505642# 116.9636993548# 93.5429378580#

H72 136.7225974560 114.9746309256 93.2913425861

H73 137.0798986862 114.4737319568 94.9309719729

H74 139.1776300668 112.8455525742 95.3873810059

H75 140.7385581080 111.5494789841 93.8960792978

H76 138.0090843430 115.0892179430 90.8795506257

H77 141.4871645707 111.4778120025 91.1563685275

H78 139.1852367419 114.3177191315 88.8502946061

H79 140.9071929443 112.5340133066 88.9791107406

C80 134.2771953704 124.5989633769 82.5231581218

C81 134.6138823088 123.8825778064 83.8219567216

O82 134.7577301203 124.4620173944 84.8929541500

H83 135.3441219956 126.4107201124 83.0714377972

C84 134.4188080022# 126.1216049453# 82.5638730531#

H85 133.5937649985# 126.6247940379# 83.0715709596#

H86 134.4691309919# 126.4689410146# 81.5301900045#

H87 133.2580342090 124.3034117337 82.2387984058

H88 134.9255548557 124.1750924542 81.7465194879

N89 136.9300557495 117.9968486760 91.3039762814

C90 135.7286045119 117.6896008374 90.8255185564

N91 134.6499348663 117.6697600710 91.6318888678

N92 135.5115937866 117.3906759070 89.5076214039

C93 136.5303856690 117.3404151973 88.5338932813

O94 136.2266924257 117.1428204358 87.3569466437

C95 137.8864236227 117.5323099169 89.0713563399

C96 137.9734369084 117.9248872683 90.4433045976

N97 138.9465350791 117.3229298219 88.2754400969

N98 139.2095598078 118.2265743041 90.9544702943

C99 140.2411789559# 117.3935380494# 88.9400339265#

C100 140.3464054672# 118.4304438593# 90.0548097265#

H101 133.7220044466 117.3730926410 91.2749454436

H102 134.8087501936 117.8578824314 92.6090686985

H103 134.5551275552 117.1560537473 89.2219809854

H104 126.2839147547 112.8511510271 93.7744492820

H105 139.1947885478 118.6931562352 91.8520609509

H106 140.4697842228 116.4027306702 89.3638844851

H107 140.3388565369 119.4480684316 89.6315789442

H108 141.2752631669 118.3062176476 90.6228427875

H109 141.0090005823 117.5971667761 88.1820686096

C110 131.8899991990# 123.9739989647# 86.7559968964#

C111 131.7158933348 122.5125130762 87.2440969183

C112 132.2885844378 122.1185495722 88.6203362553

C113 133.7714611039 121.7091929471 88.7022431729

N114 134.1181659559 120.5076514482 87.9482979372

C115 134.6053993910 120.5310528952 86.6596862617

N116 135.0013592322 121.5933564604 86.0205613932

H117 132.9134079577 124.2120138155 86.4529285831

H118 132.1106791649 121.8309423645 86.4807691388

H119 130.6388447572 122.3032874848 87.2838835064

H120 131.7060845898 121.2643418666 88.9915807762

H121 132.1236210863 122.9337681578 89.3410969520

H122 134.0211748030 121.5187756137 89.7540786988

H123 134.4334864052 122.5184258328 88.3806123994

H124 133.5975228498 119.6734352857 88.1965463161

H125 134.8126835165 122.4644051752 86.5084402716

H126 135.1860226840 119.3441794064 85.1751121915

H127 131.5752869791# 124.6768799078# 87.5273740759#

H128 131.2467799101# 124.0445940041# 85.8788530658#

N129 134.6620038166 119.2999328828 86.0405666186

H130 134.9615800398 118.5036459675 86.6068273564

O131 134.7236292379 122.5775802606 83.6339030935

H132 134.9001153714 122.0968636059 84.5424816035

H133 137.5133210570# 116.7113415011# 95.0982063077#

H134 139.0972300635 116.2575347190 94.7116540395

C135 128.9210046023# 122.2570040208# 90.4810026159#

C136 128.0389846430 121.0320828890 90.7552197362

C137 128.5259337367 120.1845586593 91.9437463665

C138 127.8416444101 118.8159400371 92.0394053523

O139 127.7260970659 118.1709577402 90.9501843842

O140 127.4646815302 118.4035099478 93.1781875392

H141 129.9385991312# 121.9481960631# 90.2378013751#

H142 128.0118665547 120.4078283636 89.8603054887

H143 127.0043233707 121.3541671391 90.9441003789

H144 128.4014279805 120.7130542734 92.8955885874

H145 129.6024095121 119.9888316047 91.8231611539

H146 128.9394991654# 122.9180979906# 91.3473970037#

H147 128.5389545633 122.8310191845 89.6283946848

C148 126.0532807340 115.4028890774 91.8273170275

N149 126.2242934147 116.0642359003 92.9587165836

H150 126.7460952155 117.0100367470 93.0342773918

H151 125.9097859692 115.6016664788 93.8033353849

N152 125.2697459895 114.1519457719 92.0707561455

O153 124.6123407648 113.7899452183 91.1260324700

N154 129.2404299295 118.3209017283 88.1654427313

C155 128.1338532180 117.9847126316 87.2902599886

C156 127.4928974230# 119.2174985242# 86.6662983411#

C157 126.9778423816 117.1563073020 87.9297874689

C158 127.4184945494 115.7590405002 88.3950891542

C159 126.4341487241 115.1047841535 89.3719548675

N160 126.4383822629 115.8388555087 90.6553094206

O161 126.6881130288 119.1866762818 85.7512933911

H162 128.8910277578 118.4693957383 89.1129873258

H163 128.5262186402 117.4168299900 86.4358445228

H164 126.1538361677 117.0695449750 87.2102408193

H165 126.6016610519 117.7383106610 88.7803691789

H166 128.4209008792 115.7952057191 88.8377733492

H167 127.5004515622 115.0944428824 87.5242861104

H168 126.7134817258 114.0647339129 89.5664515762

H169 125.4187293362 115.1068857005 88.9598841369

H170 126.9618722288 116.7609970356 90.7095401898

O171 127.8561627614 120.3920597092 87.2360785229

H172 127.3411548166 121.0664344547 86.7537258940

H173 129.8919715903 117.5344353688 88.2217435896

H174 128.2095707012 113.4666495988 92.2218949566

H175 128.8034044627 111.0619727452 98.0746088481

O176 127.0383980674 112.2399713435 93.6863378150

O177 127.4457427896 113.0578854699 91.7955479554

The first Fe-OH structure in the energy diagram in Figure 7

Energies: E= -4749,148735 solv = -0.111041, disp = -186.84 Z_0_ = 898.82

Fe1 128.0350202811 112.2436420659 95.2947007565

C2 132.0814595395 110.6394936678 91.6802178537

C3 131.5200551018 111.0618716365 90.3105923389

C4 132.6118007792# 111.2870028841# 89.2855003692#

O5 132.0893872469 111.6403499775 88.0902785820

H6 132.6074332241 109.6848187296 91.5729790975

H7 132.8425010303 111.3659637094 91.9866911575

H8 130.9368802583 111.9876531638 90.3848149227

H9 130.8234571809 110.3088663929 89.9216425451

S10 129.4221455102 112.1507288711 97.5198154532

C11 130.6681349096 112.9508181625 93.2163999786

C12 127.8891725637 115.4959689155 96.2278308720

C13 125.1016667996 111.5620042561 96.9124982935

C14 128.4571902069 108.8931005471 94.6781609804

N15 129.1231626162 113.8831868806 94.8630889661

C16 130.0836884209 114.0141490930 93.8885703471

C17 130.4274781927 115.4178310030 93.6788325869

C18 129.6892508018 116.1122571925 94.5934656644

C19 128.8733378778 115.1610389732 95.3083820785

N20 126.8112244423 113.2825243709 96.4316751766

C21 126.9088237451 114.6278618971 96.7047222922

C22 125.7616735861 115.0505869072 97.4599936110

C23 124.9602980124# 113.9563977295# 97.5889962264#

C24 125.6165253060 112.8540808500 96.9605589800

N25 127.0042263530 110.5428640527 95.7760623120

C26 125.7574245080 110.4688252343 96.3596296118

C27 125.2687993664# 109.1083985052# 96.3358988116#

C28 126.2546107127 108.3594591572 95.7590089813

C29 127.3182651867 109.2627848623 95.3904659528

N30 129.3651776345 111.1429261146 94.2481573394

C31 129.3714980122 109.7733335605 94.1116077900

C32 130.4130157005 109.3759050488 93.1969684606

C33 131.0094135666 110.5103843836 92.7248563315

C34 130.3454618412 111.6115327921 93.4049902078

H35 130.6777742582 110.3541333768 96.4456636568

H36 131.6262475176 111.1279565663 97.7667649271

H37 131.4412005871 113.1863476079 92.4946744225

H38 127.8004370006 116.5418784679 96.5024250355

H39 124.1244746598 111.3993965529 97.3562918681

H40 128.5857764901 107.8372029650 94.4601236574

H41 129.6024253346 117.1864783557 94.6906001339

H42 130.6286426744 108.3510537470 92.9189785808

H43 126.2609033844 107.2940187126 95.5654558013

H44 124.3133989389 108.7847548719 96.7280691972

H45 123.9986255569 113.8926728272 98.0836236364

H46 125.5866604721 116.0610114205 97.8050691648

C47 130.9292989205# 111.2900004587# 96.9415995103#

H48 131.3692005953# 111.9767997694# 96.2091001410#

O49 133.8031713927 111.1740741955 89.4829044259

C50 131.2805499101 115.9442753615 92.5644171650

H51 131.5856254731 116.9711494489 92.7886397450

H52 132.2059891416 115.3628102428 92.4636232216

C53 130.5164999549 115.9216600088 91.2230320177

H54 130.0875601388 114.9278618879 91.0471766111

H55 129.6628737564 116.6077243041 91.2730804704

C56 131.3489978110# 116.2862019965# 89.9720000613#

O57 132.4771762788 116.8595270815 90.1596036823

O58 130.8471102776 115.9749000861 88.8707126808

H59 132.8431424782 111.8477922375 87.5056991358

C60 138.0599986092# 116.3180010109# 94.3590002213#

C61 137.5293307014 114.8973837439 94.0241368960

C62 138.5636673308 113.9171346753 93.5365129633

C63 139.2426269730 113.0192249761 94.3227566055

C64 139.0612817723 113.7243282338 92.1935725583

N65 140.1456834436 112.2946307412 93.5593319372

C66 140.0460379528 112.6979743401 92.2428388956

C67 138.7540041986 114.3074607556 90.9510410970

C68 140.7233584991 112.2541503696 91.1005334778

C69 139.4189193934 113.8684152973 89.8113458994

C70 140.3966380813 112.8529313229 89.8877892911

H71 138.0245505429# 116.9636993774# 93.5429378768#

H72 136.7170380044 114.9803716484 93.2908412261

H73 137.0719031505 114.4774246298 94.9301282283

H74 139.1577988055 112.8357560468 95.3858683620

H75 140.7172725016 111.5364099180 93.8956104895

H76 138.0043921690 115.0887554097 90.8789401021

H77 141.4732712539 111.4686867648 91.1576214579

H78 139.1805234302 114.3152367034 88.8502303903

H79 140.9009722137 112.5300835635 88.9808975353

C80 134.2817523285 124.5985108484 82.5271162450

C81 134.6191531176 123.8880446691 83.8293487089

O82 134.7628160091 124.4728717994 84.8973702326

H83 135.3433012059 126.4136863792 83.0713735884

C84 134.4188080047# 126.1216049399# 82.5638730559#

H85 133.5937649980# 126.6247940374# 83.0715709593#

H86 134.4691309912# 126.4689410158# 81.5301900049#

H87 133.2640986988 124.2985161464 82.2421232577

H88 134.9330481608 124.1744080279 81.7530226298

N89 136.9333643673 117.9844878657 91.3103794787

C90 135.7313790966 117.6752719913 90.8335548515

N91 134.6545001171 117.6496318694 91.6418731494

N92 135.5125926794 117.3775836806 89.5155351114

C93 136.5297678902 117.3321049491 88.5400547121

O94 136.2252719805 117.1372883714 87.3628712861

C95 137.8865797784 117.5253381397 89.0757616397

C96 137.9753280965 117.9167160497 90.4476745508

N97 138.9457747012 117.3195405562 88.2775808853

N98 139.2121556275 118.2213104523 90.9570862672

C99 140.2411789932# 117.3935380703# 88.9400338976#

C100 140.3464054345# 118.4304438226# 90.0548097322#

H101 133.7234434180 117.3634059911 91.2834700157

H102 134.8110587413 117.8502270770 92.6168902375

H103 134.5550429047 117.1439621816 89.2313597978

H104 126.0842754177 112.1946122734 94.0972768908

H105 139.1965255288 118.6910558518 91.8530641522

H106 140.4748747685 116.4040324851 89.3645700420

H107 140.3340900959 119.4482815013 89.6321877387

H108 141.2770746741 118.3090473148 90.6205252440

H109 141.0070480936 117.5992683134 88.1806171376

C110 131.8899992052# 123.9739989634# 86.7559968928#

C111 131.7129032492 122.5120070911 87.2418691936

C112 132.2777751091 122.1151065519 88.6201393520

C113 133.7608574291 121.7094494819 88.7102134950

N114 134.1151727285 120.5104579427 87.9550615637

C115 134.6106275586 120.5381427400 86.6700215353

N116 135.0103239329 121.6025072703 86.0361726492

H117 132.9138830554 124.2112771573 86.4539058879

H118 132.1107633273 121.8312494322 86.4792917476

H119 130.6356759678 122.3033800104 87.2764177218

H120 131.6944005057 121.2587449990 88.9846346381

H121 132.1059823614 122.9274292748 89.3424093961

H122 134.0046390989 121.5164306700 89.7629697340

H123 134.4232364062 122.5208904287 88.3947722683

H124 133.5922348746 119.6756247777 88.1964861352

H125 134.8169244773 122.4717881931 86.5254004045

H126 135.2044025640 119.3555962622 85.1868102272

H127 131.5752869776# 124.6768799046# 87.5273740781#

H128 131.2467799079# 124.0445940045# 85.8788530675#

N129 134.6729535230 119.3087513876 86.0475526378

H130 134.9669195414 118.5104779956 86.6137461459

O131 134.7292891267 122.5822449977 83.6475849126

H132 134.9074457899 122.1051389849 84.5582239463

H133 137.5133210516# 116.7113415200# 95.0982062936#

H134 139.0969215258 116.2549282854 94.7117757031

C135 128.9210045547# 122.2570039087# 90.4810026704#

C136 128.0224002892 121.0296327760 90.7228380620

C137 128.4984823881 120.1012986564 91.8537576110

C138 127.8087363000 118.7199966595 91.8932470973

O139 127.5083368800 118.2010273882 90.7723012293

O140 127.6363964458 118.1750906072 93.0226585599

H141 129.9385991427# 121.9481961044# 90.2378013708#

H142 127.9664114124 120.4509009709 89.7991011073

H143 126.9970226003 121.3639107888 90.9399732441

H144 128.3946379419 120.5713299334 92.8389502759

H145 129.5730199125 119.8990261052 91.7194353529

H146 128.9394991674# 122.9180979922# 91.3473970023#

H147 128.5473284711 122.8413654201 89.6308551163

C148 126.7373984594 114.9176299652 91.5857754231

N149 126.8119893887 115.5483445859 92.7615792499

H150 127.0793537553 116.5697511900 92.8285591342

H151 126.9225679467 114.9710311552 93.5844115699

N152 129.2583196771 118.3324397173 88.1466078234

C153 128.2020949508 117.9910947840 87.2127198138

C154 127.4928975244# 119.2174982759# 86.6662985495#

C155 127.0856140532 117.0503470837 87.7482747934

C156 127.6006660686 115.6578896235 88.1358738343

C157 126.6807790614 114.9445972578 89.1295542978

N158 126.7263711469 115.5887225029 90.4485018493

O159 126.6520876821 119.1905563376 85.7830486171

H160 128.8507869462 118.4472103635 89.0765933835

H161 128.6581567010 117.5165885518 86.3324869069

H162 126.2934764878 116.9642766554 86.9944910777

H163 126.6465815309 117.5517998888 88.6182977965

H164 128.6102376174 115.7148559368 88.5590663894

H165 127.6771590683 115.0319865009 87.2355817125

H166 126.9783395516 113.9008572022 89.2508010187

H167 125.6419396410 114.9477696505 88.7665786756

H168 126.9339572837 116.6139612892 90.5314182489

O169 127.8457393689 120.3879789459 87.2506522510

H170 127.2836763846 121.0554374986 86.8133298517

H171 129.9216041329 117.5576687748 88.2139820033

H172 126.8267554130 113.1694599496 92.4213476315

H173 128.8065826669 111.0919413312 98.0886199927

O174 126.9702004716 112.4985075517 93.8374202573

O175 126.6349653974 113.6000735379 91.5268033301

The final Fe-OH structure in the energy diagram in Figure 6

Energies: E= -4749,161313 solv = -0.111168, disp = -186.11 Z_0_ =

Fe1 128.0677950486 112.1769467373 95.2809214136

C2 132.0957092405 110.5740093735 91.6682104705

C3 131.5323811668 111.0002627208 90.3022200559

C4 132.6118007708# 111.2870028796# 89.2855003825#

O5 132.0793397850 111.5061309487 88.0593985316

H6 132.6209008627 109.6182994591 91.5591035680

H7 132.8553558216 111.3008468799 91.9774419138

H8 130.9200820849 111.9062249045 90.3947884871

H9 130.8585250623 110.2362986209 89.8966015938

S10 129.2343041407 111.8155086487 97.3180845320

C11 130.6924391522 112.8928997580 93.2036934965

C12 127.9254913434 115.4463981808 96.2264751169

C13 125.0925564110 111.5577883746 96.8787054007

C14 128.4228800253 108.8573193205 94.6273579060

N15 129.1548551774 113.8313887211 94.8536048752

C16 130.1177862173 113.9582443204 93.8827370820

C17 130.4690667058 115.3619155446 93.6809408158

C18 129.7376904976 116.0546864288 94.6007210466

C19 128.9135608672 115.1014797313 95.3112510927

N20 126.8063050562 113.2565665488 96.4079102771

C21 126.9290412219 114.5931672644 96.6980745264

C22 125.7863400396 115.0329845784 97.4612478934

C23 124.9602980718# 113.9563977050# 97.5889962148#

C24 125.6094144733 112.8470762620 96.9425990124

N25 126.9614348895 110.5289609759 95.6775511574

C26 125.7442309600 110.4698316750 96.3063706959

C27 125.2687993865# 109.1083985253# 96.3358987965#

C28 126.2343227812 108.3440558786 95.7480879581

C29 127.2837639111 109.2430149431 95.3308071428

N30 129.3586112369 111.0883956418 94.1956854414

C31 129.3534895731 109.7230988912 94.0640056693

C32 130.4008507659 109.3152350618 93.1587451493

C33 131.0155030489 110.4462145498 92.7027032552

C34 130.3529462668 111.5529530258 93.3760990828

H35 130.9342170600 110.2830487862 96.5121868299

H36 131.5253873859 111.2991698974 97.8587068173

H37 131.4752977486 113.1247639174 92.4906173099

H38 127.8523992571 116.4930110475 96.5039702314

H39 124.1263997072 111.3810488211 97.3412855710

H40 128.5473767585 107.7968918254 94.4283741600

H41 129.6562370715 117.1288960268 94.7043192870

H42 130.6088390221 108.2892081745 92.8783026603

H43 126.2392873652 107.2731167674 95.5873485221

H44 124.3287181181 108.7906606658 96.7692943787

H45 123.9984532318 113.9029284362 98.0840434633

H46 125.6347901384 116.0452855872 97.8133399466

C47 130.9292987559# 111.2900005397# 96.9415994479#

H48 131.3692007021# 111.9767997320# 96.2091001701#

O49 133.8058370283 111.3330074077 89.4988507888

C50 131.3175786535 115.8990273364 92.5633251677

H51 131.6525823206 116.9116228108 92.8137062227

H52 132.2260313533 115.2960014526 92.4331805043

C53 130.5361413645 115.9340497064 91.2317504583

H54 130.0698255673 114.9599846591 91.0453497175

H55 129.7056203570 116.6453605751 91.3087750908

C56 131.3489978028# 116.2862020084# 89.9720000731#

O57 132.5018514306 116.8464026555 90.1373895252

O58 130.8517078581 115.9824961625 88.8702683546

H59 132.8217860446 111.7636780017 87.4810420415

C60 138.0599986327# 116.3180010073# 94.3590002094#

C61 137.5826461749 114.8804231695 94.0245032405

C62 138.6774927957 113.9534182405 93.5653036037

C63 139.4174935686 113.1310543044 94.3787257068

C64 139.2016025491 113.7671771629 92.2329464432

N65 140.3840480417 112.4641423853 93.6419845233

C66 140.2661041720 112.8239373068 92.3165412766

C67 138.8541089997 114.2811931125 90.9708441551

C68 140.9863507703 112.4028793231 91.1922060809

C69 139.5589980843 113.8570811340 89.8488116442

C70 140.6188646908 112.9309219249 89.9591819664

H71 138.0245505333# 116.9636993876# 93.5429378853#

H72 136.7825588134 114.9221108239 93.2751173403

H73 137.1262278078 114.4467054383 94.9231995764

H74 139.3351605346 112.9660634165 95.4447548946

H75 141.0013378852 111.7530947331 94.0008233629

H76 138.0326900645 114.9843040560 90.8709266917

H77 141.7944525474 111.6806748352 91.2768444995

H78 139.2728668739 114.2214147251 88.8656059487

H79 141.1475018636 112.6147023694 89.0640513120

C80 134.2708537251 124.5983059793 82.5147126209

C81 134.6043378620 123.8601255362 83.8016749807

O82 134.7806623465 124.4127472579 84.8797926153

H83 135.3451840277 126.4061395415 83.0719518055

C84 134.4188080174# 126.1216049474# 82.5638730590#

H85 133.5937649927# 126.6247940302# 83.0715709578#

H86 134.4691309859# 126.4689410137# 81.5301900039#

H87 133.2505559140 124.3100064152 82.2275902480

H88 134.9157125669 124.1782368802 81.7327857063

N89 136.8898239815 118.0114634528 91.2979220204

C90 135.6869845487 117.6746513866 90.8159668735

N91 134.6110397062 117.6387971633 91.6080310166

N92 135.4767493775 117.3535817761 89.4968098231

C93 136.5065518643 117.3064972734 88.5498877118

O94 136.2920698488 117.0493762151 87.3580082915

C95 137.8159180879 117.5864211534 89.0940315337

C96 137.9326541075 117.9705188984 90.4447644762

N97 138.9373501028 117.5290541812 88.2919331222

N98 139.1743802517 118.2937181338 90.9261146942

C99 140.2411789678# 117.3935380835# 88.9400339045#

C100 140.3464054307# 118.4304438118# 90.0548097318#

H101 133.6726427272 117.3349904073 91.2364782297

H102 134.7515399271 117.8576830899 92.5830329012

H103 134.5161948960 117.0953370126 89.2265041877

H104 126.1037203926 112.2137879960 94.0150731232

H105 139.1951652062 118.6751760098 91.8622013874

H106 140.3772368050 116.3886046296 89.3646476347

H107 140.4105823553 119.4417299578 89.6277810452

H108 141.2474445568 118.2492276773 90.6488178837

H109 141.0238418110 117.5639415479 88.1942802520

C110 131.8899992274# 123.9739989680# 86.7559968834#

C111 131.6936775609 122.5114134278 87.2362393199

C112 132.2566489790 122.0919263157 88.6086651379

C113 133.7217734886 121.6255008335 88.6833350900

N114 134.0171233393 120.4124207151 87.9200196213

C115 134.4997992666 120.4373163854 86.6291620005

N116 134.9238448916 121.4869556633 85.9904909705

H117 132.9163097722 124.2042485674 86.4569385501

H118 132.0729821184 121.8290177692 86.4656628323

H119 130.6135721608 122.3216915786 87.2746520372

H120 131.6433318309 121.2593842784 88.9777856612

H121 132.1249410115 122.9083069187 89.3341871748

H122 133.9669284858 121.4175083916 89.7328343717

H123 134.4166658648 122.4074612339 88.3627751598

H124 133.4250260909 119.6161369455 88.1363836630

H125 134.7413026336 122.3612259104 86.4764545761

H126 135.0107275155 119.2467804072 85.1171766848

H127 131.5752869709# 124.6768798967# 87.5273740826#

H128 131.2467799004# 124.0445939989# 85.8788530725#

N129 134.5235606815 119.2028353140 86.0038784458

H130 134.8357913353 118.4083934963 86.5597838036

O131 134.6750963943 122.5511991830 83.5917958442

H132 134.8394975920 122.0542452999 84.4792518016

H133 137.5133210432# 116.7113415143# 95.0982062904#

H134 139.0972906815 116.2816416125 94.7162756771

C135 128.9210045494# 122.2570038838# 90.4810026945#

C136 128.0253261025 121.0288716448 90.7265193969

C137 128.5191079034 120.0943773556 91.8428632091

C138 127.8274892506 118.7116666935 91.8850887870

O139 127.5307452664 118.1963302576 90.7600660162

O140 127.6564700529 118.1711171143 93.0123501626

H141 129.9385991432# 121.9481961126# 90.2378013623#

H142 127.9560903287 120.4554108692 89.8000080789

H143 127.0035076268 121.3620151579 90.9610312212

H144 128.4314104248 120.5587949869 92.8323289211

H145 129.5912632720 119.8929002669 91.6880146646

H146 128.9394991717# 122.9180980014# 91.3473969952#

H147 128.5451460833 122.8407042177 89.6311347239

C148 126.7757149870 114.8509372733 91.5921573617

N149 126.8430953265 115.4689585456 92.7763250274

H150 127.0918943700 116.4840784917 92.8468198339

H151 126.9753093653 114.8772374199 93.5875956076

N152 129.2572315777 118.3307542161 88.1476041524

C153 128.1927063333 117.9890388955 87.2207691567

C154 127.4928975335# 119.2174982440# 86.6662985754#

C155 127.0767520758 117.0561416597 87.7713003875

C156 127.5856012253 115.6558516198 88.1406172635

C157 126.6745638697 114.9352783202 89.1392447931

N158 126.7400690963 115.5534811982 90.4670152545

O159 126.6217793794 119.1900323542 85.8126496743

H160 128.8497366648 118.4460172702 89.0790895751

H161 128.6415619748 117.5050064103 86.3410585791

H162 126.2711209709 116.9827159606 87.0308355957

H163 126.6596640026 117.5550406554 88.6529751856

H164 128.5987576893 115.7032762819 88.5548360945

H165 127.6492863866 115.0404296774 87.2316731571

H166 126.9674258171 113.8879847565 89.2396405126

H167 125.6317179921 114.9478304217 88.7861733583

H168 126.9397397968 116.5750002048 90.5607792966

O169 127.8959235199 120.3963119657 87.2023623665

H170 127.3280035452 121.0610331345 86.7684208216

H171 129.9065563193 117.5455926640 88.2172311521

H172 126.8797030370 113.0878652355 92.4370485979

H173 138.7865966241 117.1298027811 87.3729271373

O174 126.9929235227 112.5167479501 93.7714849868

O175 126.7095064581 113.5417737494 91.5106289033

O_2_  E=-150.304600 Z_0_ = 2.38. Translational entropy is 10.8 kcal/mol

NO E=-129.869072 Z_0_ = 2.85. Translational entropy is 10.8 kcal/mol
